# Domino Aldol-S_N_Ar-Dehydration Sequence for [3+3] Annulations to Prepare Quinolin-2(1*H*)-ones and 1,8-Naphthyridin-2(1*H*)-ones

**DOI:** 10.3390/molecules28155856

**Published:** 2023-08-03

**Authors:** Kwabena Fobi, Ebenezer Ametsetor, Richard A. Bunce

**Affiliations:** Department of Chemistry, Oklahoma State University, Stillwater, OK 74078-3071, USA; kfobi@okstate.edu (K.F.); eametse@okstate.edu (E.A.)

**Keywords:** quinolin-2(1*H*)-ones, 1,8-naphthyridin-2(1*H*)-ones, heterocycle synthesis, aldol-S_N_Ar-dehydration reactions, domino reactions, [3+3] annulation

## Abstract

A domino aldol-S_N_Ar-dehydration [3+3] annulation strategy has been utilized to fuse six-membered cyclic amides onto aromatic substrates. 2-Arylacetamides have been reacted with 2-fluorobenzaldehyde derivatives activated toward S_N_Ar reaction by an electron-withdrawing substituent (NO_2_, CN, CF_3_, CO_2_Me) at C5 to prepare 3,6-disubstituted quinolin-2(1*H*)-ones. Additionally, 3-substituted 1,8-naphthyridin-2(1*H*)-ones have been similarly derived from 2-fluoronicotinaldehyde. Fifteen examples are reported, and two possible mechanistic scenarios are presented and discussed.

## 1. Introduction

Over the past 15 years, our group has reported numerous cases of domino reactions involving the S_N_Ar reaction to fuse heterocycles to pre-existing aromatic frameworks. Our early contributions in this area have been reviewed [1]. More recent projects have broadened the scope of these processes. One study described an imine addition-S_N_Ar reaction to *tert*-butyl (2-fluoro-5-nitrobenzoyl)acetate to give 4-oxo-1,2,3,4-tetrahydroquinoline-3-carboxylic esters [2]. Further use of domino procedures on Morita-Baylis-Hillman acetates afforded efficient access to naphthalenes and quinolines [3] as well as dihydroquinolines, dihydronaphthyridines and quinolin-4(1*H*)-ones [4]. Additionally, we have also reported a domino Michael-S_N_Ar-heteroaromatization sequence using S_N_Ar-activated 2-fluoroaryl-acrylate esters to prepare highly substituted 1*H*-indole-3-carboxylate esters [5].

An earlier study from this laboratory described a synthetic approach to 4*H*-1-benzo-pyrans involving a domino S_N_2 alkylation of a β-ketoester by 2-fluoro-5-nitrobenzyl bromide followed by S_N_Ar ring closure through the enolate oxygen of the alkylated product [6]. This annulation utilized a [3+3] strategy combining a double nucleophile (a β-ketoester) and a double electrophile (a benzyl bromide aryl-activated for S_N_Ar addition). The reaction was originally performed in acetone using an eight-fold excess of K_2_CO_3_, but subsequent experiments revealed that it could proceed with less base (four equiv.) in DMF solvent.

Since this report, many additional studies of [3+3] cyclizations to prepare heterocycles have been advanced. Two acid-catalyzed aza-annulations have been reported by the Hsung lab as strategies for the synthesis of alkaloids. The first described an attempt to assemble propyleine via intramolecular reaction of a ring-embedded vinylogous amide with a vinyliminium salt (Reaction (1)), but only modest results were achieved [7]. On the other hand, a chiral auxiliary was successfully used in an intermolecular variant of this reaction to generate a common precursor to two stereochemical scaffolds indigenous to the Lepadin family of alkaloids (Reaction (2)) [8].
(1)
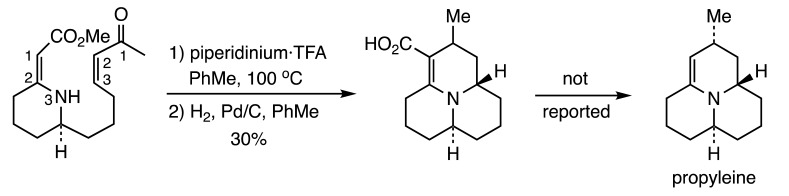
(2)
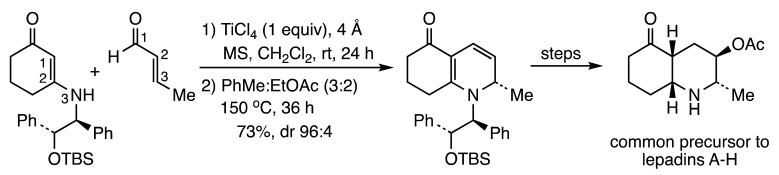


Four additional papers explored additions of nitrogen heterocycles to allylic esters. Tong and co-workers reported access to thiopyrano[2,3-*b*]indoles by addition of indoline-2-thiones to β-acetoxy allenoates (Reaction (3)) [9]. A related study by the Swamy group described the preparation of α-carbolines from β-acetoxy allenoates with iminoindolines [10] (Reaction (4)). A further investigation by the Guo lab enantioselectively produced substituted spirocyclohexenes by reaction of Morita-Baylis-Hillman carbonates with α-arylidene pyrazolinones in the presence of a chiral dihydroquinidine catalyst (Reaction (5)) [11]. The fourth investigation by Satham and Namboothiri presented a synthesis of 2-aryl terephthalates from nitroallylic acetates with stabilized sulfur ylides (Reaction (6)) [12].
(3)
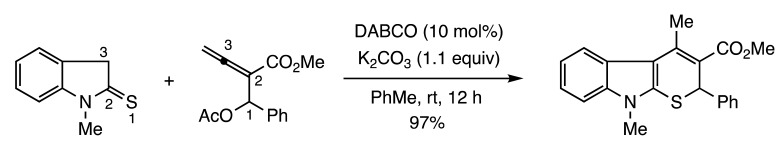
(4)
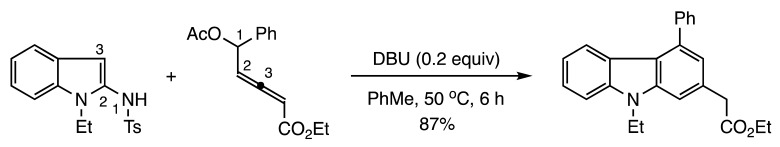
(5)
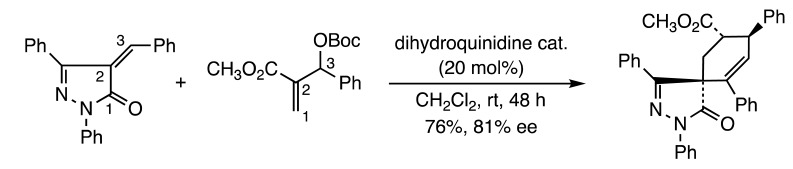
(6)
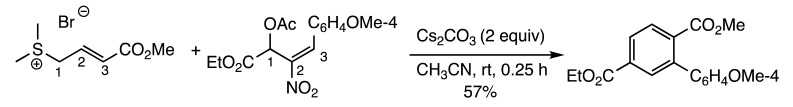


Four other articles have appeared describing metal- and organocatalyzed fusion of heterocycles to pre-existing rings. Huang and co-workers disclosed a copper-promoted synthesis of quinolines from aryl ketoximes and 2-fluorobenzaldehydes (Reaction (7)) [13]. Kanchupalli et al. unveiled a chemodivergent Rh(III)-catalyzed annulation involving indoles and iodonium carbenes to generate tri- and tetracyclic nitrogen heterocycles (Reaction (8)) [14]. The Wang group developed a zinc-catalyzed enantioselective ring formation to prepare chiral spiro[indoline-3,4′-thiopyrano[2,3-*b*]indole] derivatives [15] (Reaction (9)). Finally, the chemical synthesis team at the Sichuan Key Laboratory published a route to several chiral spiro-δ-lactam oxindoles from 3-carboxamides and β,γ-unsaturated-α-ketoesters using bifunctional urea and squaramide organocatalysts (Reaction (10)) [16]. Numerous other [3+3] annulations are detailed in a review by Feng and Liu [17].
(7)
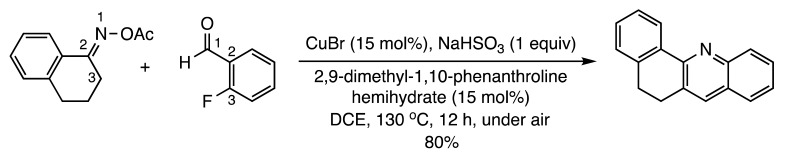
(8)
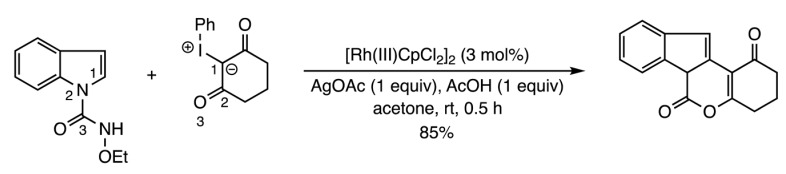
(9)
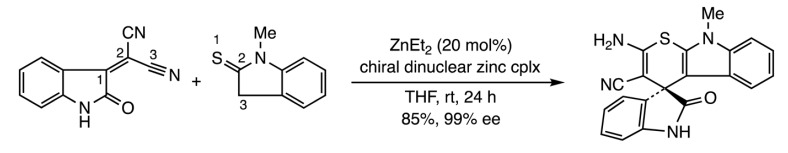
(10)
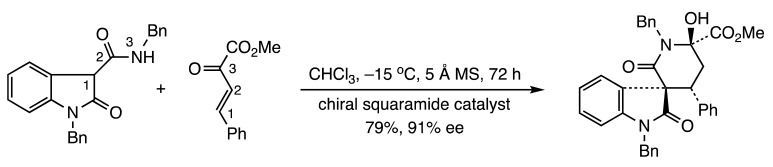


**Figure 1:**
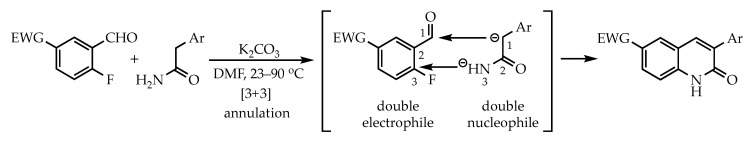
Generic [3+3] annulation leading to quinolin-2(1*H*)-ones reported in this work.

The current study reports the use of a domino aldol-S_N_Ar-dehydration reaction to prepare quinolin-2(1*H*)-ones and 1,8-naphthyridin-2(1*H*)-ones via the [3+3] annulation (Figure 1) of a double nucleophile (a 2-arylacetamide) with a double electrophile (an S_N_Ar-activated 2-fluorobenzaldehyde or 2-fluoronicotinaldehyde). The aromatic aldehydes are commercially available or easily prepared. The arylacetamides are relatively scarce and expensive to purchase but can be readily synthesized from the corresponding 2-arylacetyl chlorides. The current transformation represents the first use of an activated acetamide to construct potential drug candidates.

**Figure 2:**
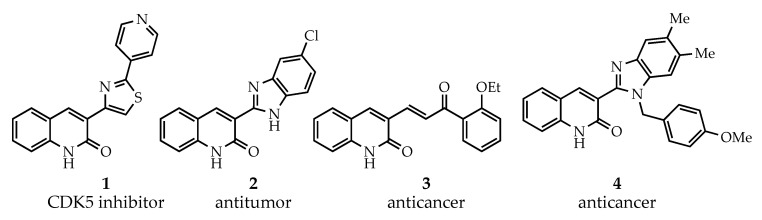
Drug candidates incorporating the 3-arylquinolin-2(1*H*)-one core.

The 3-arylquinolin-2(1*H*)-one scaffold comprises the core ring system of several important drug candidates (see Figure 2). Compound **1** is a CDK 5 inhibitor and as such may prove useful in the treatment of Alzheimer’s disease and amyotrophic lateral sclerosis [18]. The benzimidazole-linked derivative **2** slowed the growth of NCI-H460 large lung tumor cells in a xenograft mouse model [19]. The quinolone-chalcone hybrid **3** showed significant tubulin polymerization inhibition [20] and thus could find use in curbing the growth of cancer cells by interfering in the mitotic process. Finally, the benzimidazole-substituted quinolin-2(1*H*)-one **4** demonstrated promising activity against MDA-MB-231 (a model for late-stage breast cancer) and PC-3 (human prostate cancer) cell lines by decreasing CDK-1 and stabilizing the levels of Hsp90 and Hsp70 without initiating a heat shock response [21].

## 2. Results and Discussion

2-Fluoro-5-nitrobenzaldehyde (**5**) [22] and 5-carbomethoxy-2-fluorobenzaldehyde (**8**) [5] were prepared as previously described. Other benzaldehydes—5-cyano-2-fluoro-benzaldehyde (**6**), 2-fluoro-5-(trifluoromethyl)benzaldehyde (**7**), and 2-fluoronicotinaldehyde (**9**)—were commercially available. Phenylacetamide (**10**), 2-(phenylsulfonyl)acetamide (**11**), ethyl malonate monoamide (**12**), and 2-(4-methoxyphenyl)acetamide (**13**) were also acquired from a commercial source, while 2-(2-fluorophenyl)acetamide (**14**), 2-(4-chlorophenyl)acetamide (**15**), and 2-(2,5-dimethylphenyl)acetamide (**16**) were prepared from the corresponding acid chlorides [23].

A listing of the quinolin-2(1*H*)-ones and 1,8-naphthyridin-2(1*H*)-ones prepared in this study is given in Table 1. The sequence entails initial aldol addition of the carbanion derived from the 2-arylacetamide to the aldehyde carbon or S_N_Ar addition-elimination of the amide nitrogen at the fluoro-substituted carbon of the activated 2-fluorobenzaldehyde. Following bond rotation of the aldol adduct or equilibration of the amide rotamer to bring the reactive centers into proximity, heterocyclic ring closure would occur to give the ring-fused product. The entire process occurred in a single reaction vessel using K_2_CO_3_ as the base with DMF as the solvent. No other catalysts or promoters were added, and this simplified the process optimization. Four activating groups at C5 of the aromatic aldehyde, including NO_2_, CN, CF_3_, and CO_2_Me, have been validated and 2-fluoronicotinaldehyde also gave successful cyclization. Acetamides activated by aromatic or ester groups at C2 were found to be successful, while derivatives incorporating C2 cyano or ketone groups failed to successfully undergo the reaction sequence.

**Scheme 1:**
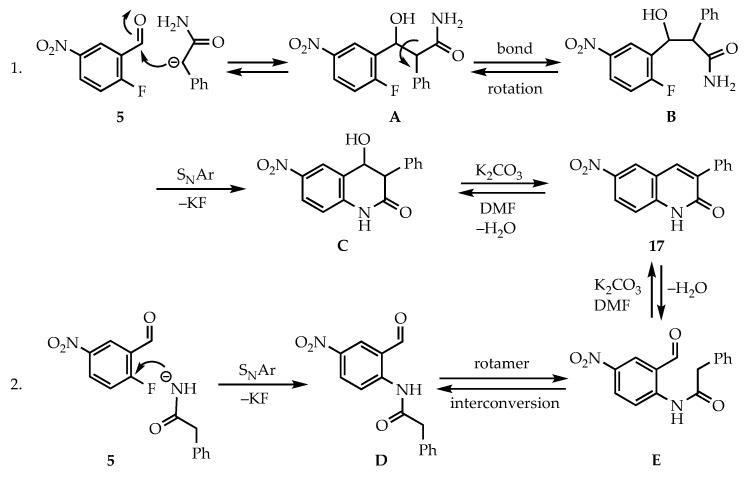
Plausible mechanisms for the formation of **17** from **5** and the anion of phenylacetamide (**10**).

As briefly mentioned above, two mechanisms for the formation of the target heterocycles are plausible, differing in the initial bond-forming step (see Scheme 1). Since the pKa values for the pseudo-acidic methylene and amido groups of the 2-arylacetamides are comparable, it might be expected that the anion from either site could initiate the sequence. These pKa’s were estimated from experimentally derived values for Ph**CH_2_**CO_2_Et (22.6), PhCO**NH_2_** (23.3), CH_3_CO**NH_2_** (25.5) and PhCH_2_CONH_2_ (unclearly listed as 24.7) in DMSO [24]. Computer-estimated pKa’s were similar but trended slightly more acidic than the experimental values [25]. Mechanism 1 requires an initial aldolization between the arylacetamide methylene anion and the benzaldehyde to yield aldol conformers **A** (possibly H-bond stabilized) and **B**. S_N_Ar ring closure from conformation **B** would then give the fused-ring β-hydroxyamide, which upon loss of water would afford the quinolin-2(1*H*)-one. Interestingly, the ring closure in this sequence could occur from either the enolate oxygen or the amide nitrogen. However, as one might expect, cyclization by nitrogen prevails since the quinolin-2(1*H*)-one product has greater stability than the alternative 2*H*-chromen-2-imine. For quinolin-2(1*H*)-ones, it is well known that the amide structure predominates over the quinolinol tautomer [26,27], and with its 10 π electron complement and planarity (all atoms are sp^2^ hybridized) retains its aromaticity [28]. A similar preference likely applies in the 1,8-naphthyridin-2(1*H*)-one series. Mechanism 2, involving the addition of the amide nitrogen anion to the aromatic ring, offers an alternative route to the final products. We have shown in the past that primary amides give high yields of S_N_Ar products in DMF [29], but usually require heat for efficient reaction. In this scenario, the less hindered amide rotamer **D** would be expected to predominate, necessitating a conversion to the more sterically challenged rotamer **E** before ring formation could occur. To gain insight into the initiating reaction of the sequence, two reactions were run on substrates bearing similar functionality to those used to prepare quinolin-2(1*H*)-ones. The first, between phenylacetamide and 2-nitrobenzaldehyde in DMF with K_2_CO_3_ at room temperature (23 °C), formed two inseparable polar products (by TLC) with loss of the aldehyde carbonyl (IR). The second, employing phenylacetamide with methyl 2-fluoro-5-nitrobenzoate under the same conditions, did not show significant conversion until heat (>50 °C) was applied. While these observations do not have direct relevance in the current application, they strongly suggest that the aldehyde is the most reactive functional group. Since the reaction is stirred at 23 °C for 15–30 min prior to the application of heat, the sequence of events likely begins with aldol formation as in mechanism 1.

## 3. Materials and Methods

### 3.1. General Methods

Unless otherwise indicated, all reactions were performed under dry N_2_ in oven-dried glassware. All reagents and solvents were used as received. All wash solutions in work-up procedures were aqueous. Reactions were monitored by thin layer chromatography on Analtech No 21521 silica gel GF plates (Newark, DE, USA). Preparative separations were performed by flash chromatography on Davisil^®^, grade 62, 60–200 mesh silica gel containing 0.5% of UV-05 phosphor (both from Sorbent Technologies, Norcross, GA, USA) slurry packed into quartz columns. Band elution for all chromatographic separations was monitored using a hand-held UV lamp (Fisher Scientific, Pittsburgh, PA, USA). Melting points were obtained using a MEL-TEMP apparatus (Cambridge, MA, USA) and are uncorrected. FT-IR spectra were run as thin films on NaCl disks using a Nicolet iS50 spectrophotometer (Madison WI, USA). ^1^H- and ^13^C-NMR spectra were measured using a Bruker Avance 400 system (Billerica, MA, USA) at 400 MHz and 101 MHz, respectively, in the indicated solvents containing 0.05% (CH_3_)_4_Si as the internal standard; coupling constants (*J*) are given in Hz. Low-resolution mass spectra were obtained using a Hewlett-Packard Model 1800A GCD GC-MS system (Palo Alto, CA, USA). Elemental analyses (±0.4%) on all new compounds were determined by Atlantic Microlabs (Norcross, GA, USA). Copies of ^1^H-NMR and ^13^C-NMR spectra for all new compounds are given in the Supplementary Materials.

2-Fluoro-5-nitrobenzaldehyde (**5**) [22] and 5-carbomethoxy-2-fluorobenzaldehyde (**8**) [5] were prepared as previously described. 5-Cyano-2-fluorobenzaldehyde (**6**), 2-fluoro-5-(trifluoromethyl)benzaldehyde (**7**), 2-fluoronicotinaldehyde (**9**), phenylacetamide (**10**), 2-(phenylsulfonyl)acetamide (**11**), ethyl malonate monoamide (**12**), 2-(4-methoxyphenyl)-acetamide (**13**), 2-(2-fluorophenyl)acetyl chloride, 2-(4-chlorophenyl)acetyl chloride, and 2-(2,5-dimethylphenyl)acetyl chloride were purchased from Combi Blocks, Inc. (San Diego, CA, USA).

### 3.2. General Procedure for the Preparation of 2-Arylacetamides

The procedure of Finan and Fothergill was adapted [23]. To a solution of ammonium acetate [ground to a powder and dried under high vacuum (ca. 0.5 mmHg) for 2 h at 23 °C, 1.82 equiv.] in dry acetone (25 mL) was added 1 equiv. of the 2-arylacetyl chloride. The mixture was stirred for 3 h and filtered through Celite^®^ with acetone (50–100 mL). The acetone solution was concentrated, and the resulting solid was triturated with ether in hexane (5:1 *v*/*v*). The solid was collected by filtration, recrystallized from water for **14** and **15** (or 19:1 *v*/*v* water-ethanol for **16**) and dried at 23 °C under a high vacuum to give the yields reported.

#### 3.2.1. 2-(2-Fluorophenyl)acetamide (**14**)

From 2-(2-fluorophenyl)acetyl chloride, yield 0.89 g (65%) as a white solid, m.p. 156–157 °C (lit. [30] m.p. 157.2–159.2 °C); IR: 3389, 3202, 1645, 1626, 1227 cm^−1^; ^1^H NMR (400 MHz, CDCl_3_): δ_H_ 7.34–7.27 (complex, 2H), 7.14 (td, *J* = 7.6, 1.2 Hz, 1H), 7.09 (ddd, *J* = 9.6, 8.1, 1.2 Hz, 1H), 5.59 (br s, 1H), 5.51 (br s, 1H), 3.61 (d, *J* = 1.4 Hz, 2H); ^13^C NMR (101 MHz, CDCl_3_): δ_C_ 172.2, 160.9 (d, *J* = 245.8 Hz), 131.6 (d, *J* = 3.9 Hz), 129.4 (d, *J* = 8.2 Hz), 124.6 (d, *J* = 3.6 Hz), 122.0 (d, *J* = 15.9 Hz), 115.7 (d, *J* = 21.7 Hz), 36.4 (d, *J* = 3.0 Hz); MS (*m/z*) 153 (M^+·^).

#### 3.2.2. 2-(4-Chlorophenyl)acetamide (**15**)

From 2-(4-chlorophenyl)acetyl chloride, yield 0.89 g (66%) as a white solid, m.p. 175–176 °C (lit. [31] m.p. 175 °C); IR: 3405, 3204, 1650, 1620 cm^−1^; ^1^H NMR (400 MHz, DMSO-*d_6_*): δ_H_ 7.48 (br s, 1H), 7.36 (d, *J* = 9.0 Hz, 2H), 7.28 (d, *J* = 9.0 Hz, 2H), 6.91 (br s, 1H), 3.37 (s, 2H); ^13^C NMR (101 MHz, DMSO-*d_6_*): δ_C_ 172.3, 136.0, 131.5, 131.4, 128.5, 41.8; MS (*m/z*) 168, 170 (M^+·^, ca 3:1).

#### 3.2.3. 2-(2,5-Dimethylphenyl)acetamide (**16**)

From 2-(2,5-dimethylphenyl)acetyl chloride, yield 0.91 g (68%) as a white solid, m.p. 152–154 °C (lit. [32] m.p. 154 °C); IR: 3390, 3188, 1645, 1621 cm^−1^; ^1^H NMR (400 MHz, DMSO-*d_6_*): δ_H_ 7.34 (br s, 1H), 7.01 (d, *J* = 7.6 Hz, 1H), 6.99 (d, *J* = 1.9 Hz, 1H), 6.92 (dd, *J* = 7.6, 1.9 Hz, 1H), 6.86 (br s, 1H), 3.35 (s, 2H), 2.23 (s, 3H), 2.19 (s, 3H); ^13^C NMR (101 MHz, DMSO-*d_6_*): δ_C_ 172.6, 135.3, 134.8, 133.8, 131.2, 130.1, 127.4, 40.4, 21.0, 19.3; MS (*m/z*) 163 (M^+·^).

### 3.3. Representative Procedure for the Preparation of Quinolin-2(1H)-ones and 1,8-Naphthyridin-2(1H)-ones

A. For amides with a phenyl, phenylsulfonyl, or ester at C2 (substrates **10–12**): To a solution of the aldehyde (2.0 mmol, 2.0 equiv.) in DMF (2 mL) was added the amide (1.0 mmol, 1.0 equiv.) and K_2_CO_3_ (2.0 equiv.) at 23 °C and the mixture was stirred for 15–30 min. The reaction was then heated for 3–5 h at 90 °C. B. For amides with a substituted aromatic group at C2 (substrates **13–16**): To a solution of the aldehyde (1.0 mmol, 1.0 equiv.) in DMF (2 mL) was added the amide (2.0 mmol, 2.0 equiv.) and K_2_CO_3_ (2.0 equiv.) using the same time and temperature regime. After TLC (10–20% *v*/*v* EtOAc/hexane) indicated the reaction was complete, the crude mixture was cooled to 23 °C, poured into water (30 mL) and the aqueous layer was extracted with EtOAc (3 × 25 mL). The organic layer was washed with 1.0 M HCl (2 × 25 mL) and saturated NaHCO_3_ (30 mL). The combined organic layers were washed with saturated NaCl (30 mL) and dried (Na_2_SO_4_). Removal of the solvent under vacuum gave a crude product, which was further purified by column chromatography and crystallization from ether.

#### 3.3.1. 6-Nitro-3-phenylquinolin-2(1*H*)-one (**17**)

Yield: 0.41 g (91%) as a white solid, m.p. 299–300 °C (lit. [33] m.p. 299–302 °C); IR: 1672, 1540, 1336 cm^−1^; ^1^H NMR (400 MHz, CDCl_3_): δ_H_ 12.51 (br s, 1H), 8.77 (d, *J* = 2.2 Hz, 1H), 8.36 (s, 1H), 8.35 (dd, *J* = 9.1, 2.2 Hz, 1H), 7.76 (d, *J* = 9.1 Hz, 2H), 7.49–7.39 (complex, 4H); ^13^C NMR (101 MHz, CDCl_3_): δ_C_ 161.7, 143.1, 142.1, 137.8, 135.8, 133.9, 129.2, 128.9, 128.6, 125.3, 125.0, 119.5, 116.2; MS (*m/z*): 266 (M^+·^).

#### 3.3.2. 6-Nitro-3-(phenylsulfonyl)quinolin-2(1*H*)-one (**18**)

Yield: 0.35 g (89%) as a white solid, m.p. 329–330 °C; IR: 3160, 1662, 1625, 1338, 1159 cm^−1^; ^1^H NMR (400 MHz, DMSO-*d_6_*): δ_H_ 12.77 (s, 1H), 9.24 (s, 1H), 9.09 (d, *J* = 2.2 Hz, 1H), 8.47 (d, *J* = 9.1 Hz, 1H), 8.02 (d, *J* = 7.7 Hz, 2H), 7.74 (t, *J* = 7.4 Hz, 1H), 7.64 (t, *J* = 7.7 Hz, 2H), 7.49 (d, *J* = 9.1 Hz, 1H); ^13^C NMR (101 MHz, DMSO-*d_6_*): δ_C_ 157.0, 145.42, 145.37, 142.6, 139.6, 134.5, 133.1, 129.5, 129.0, 128.4, 127.7, 117.23, 117.16; MS (*m/z*): 330 (M^+·^). Anal. Calcd for C_15_H_10_N_2_O_5_S: C, 54.54; H, 3.05; N, 8.48. Found: C, 54.43; H, 3.01; N, 8.40.

#### 3.3.3. 3-Carbethoxy-6-nitroquinolin-2(1*H*)-one (**19**)

Yield: 0.28 g (90%) as a white solid, m.p. 187–188 °C; IR: 3112, 1689, 1619, 1526, 1348 cm^−1^; ^1^H NMR (400 MHz, CDCl_3_): δ_H_ 8.58 (s, 1H), 8.57 (d, *J* = 2.6 Hz, 1H), 8.50 (dd, *J* = 9.1, 2.6 Hz, 1H), 7.51 (d, *J* = 9.1 Hz, 1H), 4.45 (q, *J* = 7.1 Hz, 2H), 1.43 (t, *J* = 7.1 Hz, 3H); ^13^C NMR (101 MHz, CDCl_3_): δ_C_ 162.1, 158.4, 154.9, 146.9, 144.3, 128.6, 125.2, 120.6, 118.1, 117.8, 62.6, 14.2; MS (*m/z*): 262 (M^+·^). Anal. Calcd for C_12_H_10_N_2_O_5_: C, 54.97; H, 3.84; N, 10.68. Found: C, 55.01; H, 3.85; N, 10.62.

#### 3.3.4. 3-(4-Methoxyphenyl)-6-nitroquinolin-2(1*H*)-one (**20**)

Yield: 0.49 g (90%) as a white solid, m.p. 219–220 °C; IR: 3116, 2846, 1668, 1625, 1525, 1353 cm^−1^; ^1^H NMR (400 MHz, DMSO-*d_6_*): δ_H_ 8.75 (d, *J* = 2.7 Hz, 1H), 8.40 (dd, *J* = 9.1, 2.7 Hz, 1H), 8.39 (s, 1H), 7.73 (d, *J* = 8.8 Hz, 2H), 7.66 (d, *J* = 9.1 Hz, 1H), 7.07 (d, *J* = 8.8 Hz, 2H), 3.82 (s, 3H), (NH exchanged); ^13^C NMR (101 MHz, DMSO-*d_6_*): δ_C_ 160.5, 159.4, 156.8, 144.1, 138.3, 130.4, 128.6, 126.6, 126.3, 124.6, 120.5, 117.8, 114.3, 55.7; MS (*m/z*): 296 (M^+·^). Anal. Calcd for C_16_H_12_N_2_O_4_: C, 64.86; H, 4.08; N, 9.46. Found: C, 64.79; H, 4.04; N, 9.39.

#### 3.3.5. 3-(2-Fluorophenyl)-6-nitroquinolin-2(1*H*)-one (**21**)

Yield: 0.30 g (89%) as a white solid, m.p. 324–325 °C; IR: 3153, 1671, 1929, 1543, 1244, 1344 cm^−1^; ^1^H NMR (400 MHz, DMSO-*d_6_*): δ_H_ 12.56 (s, 1H), 8.77 (d, *J* = 2.6 Hz, 1H), 8.38 (dd, *J* = 9.0, 2.6 Hz, 1H), 8.28 (s, 1H), 7.50 (overlapping t, *J* = 7.8 Hz, 1H) and d, *J* = 9.0 Hz, 2H), 7.30 (m, 2H); ^13^C NMR (101 MHz, DMSO-*d_6_*): δ_C_ 160.9, 160.1 (d, *J* = 246.9 Hz), 143.5, 142.2, 140.0, 132.1 (d, *J* = 3.3 Hz), 131.0 (d, *J* = 8.4 Hz), 130.2, 125.8, 125.1, 124.7 (d, *J* = 3.5 Hz), 123.9 (d, *J* = 14.9 Hz), 119.0, 116.4, 116.1 (d, *J* = 21.6 Hz); MS (*m/z*): 284 (M^+·^). Anal. Calcd for C_15_H_9_FN_2_O_3_: C, 63.38; H, 3.19; N, 9.86. Found: C, 63.35; H, 3.17; N, 9.81.

#### 3.3.6. 3-(4-Chlorophenyl)-6-nitroquinolin-2(1*H*)-one (**22**)

Yield: 0.47 g (88%) as a white solid, m.p. 210–211 °C; IR: 3116, 1662, 1625, 1530, 1340 cm^−1^; ^1^H NMR (400 MHz, DMSO-*d_6_*): δ_H_ 8.76 (d, *J* = 2.7 Hz, 1H), 8.48 (s, 1H), 8.44 (dd, *J* = 9.1, 2.7 Hz, 1H), 7.78 (d, *J* = 8.6 Hz, 2H), 7.68 (d, *J* = 9.1 Hz, 1H), 7.59 (d, *J* = 8.6 Hz, 2H), (NH exchanged); ^13^C NMR (101 MHz, DMSO-*d_6_*): δ_C_ 159.2, 157.1, 144.1, 140.1, 134.4, 133.3, 130.8, 128.9, 128.0, 126.8, 124.9, 120.2, 118.0; MS (*m/z*): 299, 301 (M^+·^, *ca* 3:1). Anal. Calcd for C_15_H_9_ClN_2_O_3_: C, 59.92; H, 3.02; N, 9.32. Found: C, 59.88; H, 2.98; N, 9.30.

#### 3.3.7. 3-(2,5-Dimethylphenyl)-6-nitroquinolin-2(1*H*)-one (**23**)

Yield: 0.47 g (86%) as a white solid, m.p. 217–218 °C; IR: 3112, 1661, 1623, 1524, 1349 cm^−1^; ^1^H NMR (400 MHz, DMSO-*d_6_*): δ_H_ 8.76 (d, *J* = 2.8 Hz, 1H), 8.45 (dd, *J* = 9.1, 2.8 Hz, 1H), 8.20 (s, 1H), 7.69 (d, *J* = 9.1 Hz, 1H), 7.20 (d, *J* = 7.8 Hz, 1H), 7.17 (dd, *J* = 7.8, 1.8 Hz, 1H), 7.12 (d, *J* = 1.8 Hz, 1H), 2.31 (s, 3H), 2.19 (s, 3H), (NH exchanged); ^13^C NMR (101 MHz, DMSO-*d_6_*): δ_C_ 159.1, 157.4, 144.1, 141.7, 135.2, 134.5, 133.7, 130.8, 130.7, 130.5, 129.9, 126.7, 124.8, 120.1, 118.9, 20.9, 19.5; MS (*m/z*): 294 (M^+·^). Anal. Calcd for C_17_H_14_N_2_O_3_: C, 68.91; H, 5.44; N, 9.45. Found: C, 68.86; H, 5.44; N, 9.42.

#### 3.3.8. 6-Cyano-3-phenylquinolin-2(1*H*)-one (**24**)

Yield: 0.28 g (85%) as a white solid, m.p. 316–317 °C; IR: 3141, 2227, 1652 cm^−1^; ^1^H NMR (400 MHz, DMSO-*d_6_*): δ_H_ 12.35 (s, 1H), 8.28 (d, *J* = 1.9 Hz, 1H), 8.15 (s, 1H), 7.88 dd, *J =* 8.6, 1.9 Hz, 1H), 7.74 (d, *J* = 7.1 Hz, 2H), 7.49–7.38 (complex, 4H); ^13^C NMR (101 MHz, DMSO-*d_6_*): δ_C_ 161.5, 141.5, 137.1, 136.0, 133.72, 133.66, 133.1, 129.2, 128.8, 128.5, 120.1, 119.4, 116.4, 104.4; MS (*m/z*): 246 (M^+·^). Anal. Calcd for C_16_H_10_N_2_O: C, 78.03; H, 4.09; N, 11.38. Found: C, 77.95; H, 4.06; N, 11.25.

#### 3.3.9. 6-Cyano-3-(phenylsulfonyl)quinolin-2(1*H*)-one (**25**)

Yield: 0.38 g (91%) as a white solid, m.p. 357–358 °C; IR: 3149, 2230, 1656, 1622, 1322, 1158 cm^−1^; ^1^H NMR (400 MHz, DMSO-*d_6_*): δ_H_ 12.63 (s, 1H), 9.04 (s, 1H), 8.61 (s, 1H), 8.05–7.95 (complex, 3H), 7.73 (t, *J* = 7.5 Hz, 1H), 7.63 (t, *J* = 7.6 Hz, 2H), 7.45 (d, *J* = 8.7 Hz, 1H); ^13^C NMR (101 MHz, DMSO-*d_6_*): δ_C_ 156.9, 144.5, 144.0, 139.6, 136.5, 136.1, 134.4, 132.8, 129.5, 129.0, 118.8, 117.7, 117.2, 105.4; MS (*m/z*): 310 (M^+·^); Anal. Calcd for C_16_H_10_N_2_O_3_S: C, 61.93; H, 3.25; N, 9.08. Found: C, 61.90; H, 3.27; N, 9.05.

#### 3.3.10. 6-Carbomethoxy-3-phenylquinolin-2(1*H*)-one (**26**)

Yield: 0.29 g (93%) as a white solid, m.p. 254–255 °C; IR: 3155, 1726, 1654, 1621 cm^−1^; ^1^H NMR (400 MHz, DMSO-*d_6_*): δ_H_ 12.27 (s, 1H), 8.42 (d, *J* = 2.0 Hz, 1H), 8.27 (s, 1H), 8.05 (dd, *J* = 8.6, 2.0 Hz, 1H), 7.76 (d, *J* = 7.7 Hz, 2H), 7.45 (t, *J* = 7.7 Hz, 2H), 7.40 (d, *J* = 8.6 Hz, 2H), 3.88 (s, 3H); ^13^C NMR (101 MHz, DMSO-*d_6_*): δ_C_ 166.2, 161.7, 142.0, 138.2, 136.3, 132.9, 130.9, 130.7, 129.2, 128.6, 128.5, 123.5, 119.6, 115.5, 52.6; MS (*m/z*): 279 (M^+·^); Anal. Calcd for C_17_H_13_NO_3_: C, 73.11; H, 4.69; N, 5.02. Found: C, 73.04; H, 4.65; N, 4.96.

#### 3.3.11. 6-Carbomethoxy-3-(phenylsulfonyl)quinolin-2(1*H*)-one (**27**)

Yield: 0.34 g (90%) as a white solid, m.p. 318–319 °C; IR: 3151, 1721, 1662, 1625 cm^−1^; ^1^H NMR (400 MHz, DMSO-*d_6_*): δ_H_ 12.55 (s, 1H), 9.14 (s, 1H), 8.71 (d, *J* = 2.0 Hz, 1H), 8.18 (dd, *J* = 8.7, 2.0 Hz, 1H), 8.00 (d, *J* = 7.5 Hz, 2H), 7.71 (tt, *J* = 7.5, 1.2 Hz, 1H), 7.63 (t, *J* = 8.0 Hz, 2H), 7.42 (d, *J* = 8.7 Hz, 1H), 3.89 (s, 3H); ^13^C NMR (101 MHz, DMSO-*d_6_*): δ_C_ 165.8, 157.0, 145.6, 144.4, 139.9, 134.3, 134.2, 133.3, 132.1, 129.5, 128.9, 124.4, 117.3, 116.4, 52.8; MS (*m/z*): 343 (M^+·^); Anal. Calcd for C_17_H_13_NO_5_S: C, 59.47; H, 3.82; N, 4.08. Found: C, 59.49; H, 3.83; N, 4.04.

#### 3.3.12. 3-Phenyl-6-(trifluoromethyl)quinolin-2(1*H*)-one (**28**)

Yield: 0.27 g (91%) as a white solid, m.p. 234–235 °C; IR: 3152, 1654, 1637, 1331, 1156, 1103 cm^−1^; ^1^H NMR (400 MHz, CDCl_3_): δ_H_ 12.20 (s, 1H), 7.96 (s, 1H), 7.90 (t, *J* = 1.2 Hz, 1H), 7.80 (dt, *J* = 6.8, 1.7 Hz, 2H), 7.69 (dd, *J* = 8.7, 2.0 Hz, 1H), 7.54–7.43 (complex, 4H); ^13^C NMR (101 MHz, DMSO-*d_6_*): δ_C_ 166.4, 145.9, 142.4, 140.9, 138.2, 133.9, 133.4, 133.3, 131.5 (q, *J* = 3.5 Hz), 130.9 (q, *J* = 4.3 Hz), 127.5 (q, *J* = 32.3 Hz), 124.4, 124.2 (q, *J* = 272.5 Hz), 120.9; MS (*m/z*): 289 (M^+·^). Anal. Calcd for C_16_H_10_FNO: C, 66.44; H, 3.48; N, 4.84. Found: C, 66.38; H, 3.45; N, 4.81.

#### 3.3.13. 3-(Phenylsulfonyl)-6-(trifluoromethyl)quinolin-2(1*H*)-one (**29**)

Yield: 0.35 g (95%) as a white solid, m.p. 319–320 °C; IR: 3160, 1659, 1634, 1333, 1168, 1133 cm^−1^; ^1^H NMR (400 MHz, DMSO-*d_6_*): δ_H_ 12.57 (s, 1H), 9.14 (s, 1H), 8.55 (d, *J* = 2.2 Hz, 1H), 8.05–7.95 (complex, 3H), 7.74 (t, *J* = 7.5 Hz, 1H), 7.63 (t, *J* = 7.7 Hz, 2H), 7.51 (d, *J* = 8.7 Hz, 1H); ^13^C NMR (101 MHz, DMSO-*d_6_*): δ_C_ 157.0, 145.1, 143.7, 139.7, 134.4, 132.6, 130.2 (q, *J* = 3.8 Hz), 129.5, 129.0, 124.5 (q, *J* = 271.9 Hz), 123.7, 123.3, 117.3, 117.1; MS (*m/z*): 353 (M^+·^). Anal. Calcd for C_16_H_10_F_3_NO_3_S: C, 54.39; H, 2.85; N, 3.96. Found: C, 54.33; H, 2.87; N, 3.95.

#### 3.3.14. 3-Phenyl-1,8-naphthyridin-2(1*H*)-one (**30**)

Yield: 0.32 g (89%) as a white solid, m.p. 242–243 °C; IR: 3164, 1658, 1609 cm^−1^; ^1^H NMR (400 MHz, CDCl_3_): δ_H_ 11.99 (s, 1H), 8.77 (dd, *J* = 4.9, 1.7 Hz, 1H), 7.97 (dd, *J* = 7.7, 1.7 Hz, 1H), 7.84 (s, 1H), 7.76 (d, *J* = 7.5 Hz, 2H), 7.50–7.39 (complex, 3H), 7.24 (dd, *J* = 7.7, 4.9 Hz, 1H); ^13^C NMR (101 MHz, CDCl_3_): δ_C_ 162.4, 149.9, 149.3, 136.3, 136.0, 135.4, 134.6, 128.9, 128.6, 128.4, 118.7, 115.6; MS (*m/z*): 222 (M^+·^). Anal. Calcd for C_14_H_10_N_2_O: C, 75.66; H, 4.54; N, 12.60. Found: C, 75.59; H, 4.51; N, 12.51.

#### 3.3.15. 3-(Phenylsulfonyl)-1,8-naphthyridin-2(1*H*)-one (**31**)

Yield: 0.43 g (93%) as a white solid, m.p. 299–300 °C; IR: 3146, 1651, 1608, 1310, 1149 cm^−1^; ^1^H NMR (400 MHz, DMSO-*d_6_*): δ_H_ 12.71 (s, 1H), 9.03 (s, 1H), 8.68 (dd, *J* = 4.7, 1.8 Hz), 8.49 (dd, *J* = 7.9, 1.8 Hz, 1H), 8.02 (d, *J* = 7.5 Hz, 2H), 7.72 (tt, *J* = 7.5, 1.3 Hz, 1H), 7.62 (t, *J* = 7.5 Hz, 2H), 7.38 (dd, *J* = 7.9, 4.7 Hz, 1H); ^13^C NMR (101 MHz, DMSO-*d_6_*): δ_C_ 156.6, 153.5, 150.6, 143.6, 139.0, 138.7, 133.2, 131.2, 128.4, 127.9, 118.7, 112.0; MS (*m/z*): 286 (M^+·^). Anal. Calcd for C_14_H_10_N_2_O_3_.S: C, 58.73; H, 3.53; N, 9.78. Found: C, 58.68; H, 3.52; N, 9.71.

## 4. Conclusions

We have successfully developed a domino aldol-S_N_Ar-dehydration [3+3] annulation for the synthesis of 3,6-disubstituted quinolin-2(1*H*)-ones and 3-substituted 1,8-naphthyridin-2(1*H*)-ones. The transformation involves an aldol reaction of activated acetamides, substituted at C2 by an aryl, phenylsulfonyl, or ester group with 2-fluorobenzaldehydes substituted with electron-withdrawing substituents at C5 to promote S_N_Ar reaction. The process was also found to be successful for 2-fluoronicotinaldehyde. Both target fused heterocycles were produced in high yields by this process. Since the methylene and amido protons of the amide reacting partner have similar pKa values, two different mechanisms, differing in the initial attacking anion, were possible. Model reactions of phenylacetamide with 2-nitrobenzaldehyde and methyl 2-fluoro-5-nitrobenzoate in DMF with K_2_CO_3_ at 23 °C suggested that aldol addition of the phenylacetamide methylene to the aldehyde triggers the cascade of events. The current work represents the first use of a 2-arylacetamide in a potentially valuable synthetic application.

## Figures and Tables

**Table 1:** Synthesis of quinolin-2(1*H*)-one and 1,8-naphthyridin-2(1*H*)-one derivatives.

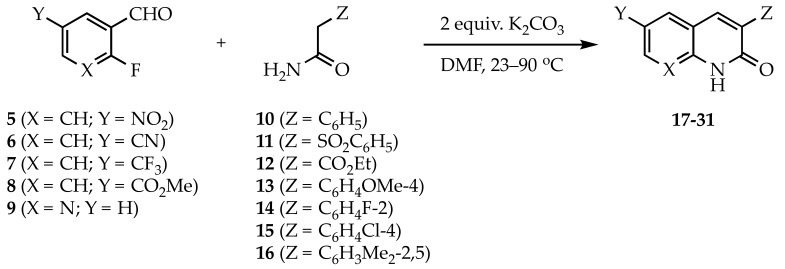				
Aldehyde	Amide	Product	Product No.	Yield (%) ^a^
**5**	**10**	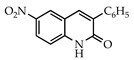	**17**	91
**5**	**11**	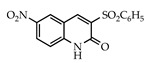	**18**	89
**5**	**12**	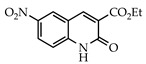	**19**	90
**5**	**13**	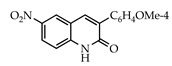	**20**	90
**5**	**14**	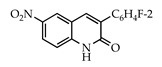	**21**	89
**5**	**15**	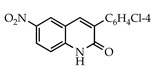	**22**	88
**5**	**16**	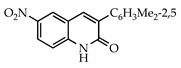	**23**	86
**6**	**10**	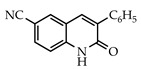	**24**	85
**6**	**11**	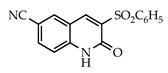	**25**	91
**7**	**10**	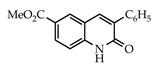	**26**	93
**7**	**11**	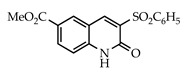	**27**	90
**8**	**10**	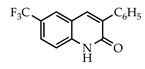	**28**	91
**8**	**11**	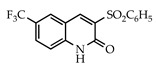	**29**	95
**9**	**10**	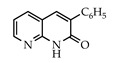	**30**	89
**9**	**11**	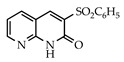	**31**	90

^a^ Yields are isolated.

## Data Availability

Not applicable.

## Supplementary Materials

The following supporting information can be downloaded at: https://www.mdpi.com/article/10.3390/molecules28155856/s1, Copies of ^1^H-NMR and ^13^C-NMR spectra for all new compounds are available online.
