# Supplementary material for: Domino Aldol-SNAr-Dehydration Sequence for [3+3] Annulations to Prepare Quinolin-2(1H)-ones and 1,8-Naphthyridin-2(1H)-ones
Source: Molecules. 2023 Aug 3;28(15):5856. doi: 10.3390/molecules28155856 (PMC10420682; doi:10.3390/molecules28155856)

Domino Aldol-SNAr-Dehydration Sequence for [3+3] Annulations to Prepare Quinolin-2(1*H*)-ones and 1,8-Naphthyridin-2(1*H*)-ones

Kwabena Fobi, Ebenezer Ametsetor and Richard A. Bunce *

Department of Chemistry, Oklahoma State University, Stillwater, OK, 74078-3071, USA
* Correspondence: rab@okstate.edu; Tel.: +1-405-744-5952

Supplementary Information

Compound **page**

^{1}H NMR for 2-(2-Fluorophenyl)acetamide (**14**) 3
^{13}C NMR for 2-(2-Fluorophenyl)acetamide (**14**) 3

^{1}H NMR for 2-(4-Chlorophenyl)acetamide (**15**) 4
^{13}C NMR for 2-(4-Chlorophenyl)acetamide (**15**) 4

^{1}H NMR for 2-(2,5-Dimethylphenyl)acetamide (**16**) 5
^{13}C NMR for 2-(2,5-Dimethylphenyl)acetamide (**16**) 5

^{1}H NMR for 6-Nitro-3-phenylquinolin-2(1*H*)-one (**17**) 6
^{13}C NMR for 6-Nitro-3-phenylquinolin-2(1*H*)-one (**17**) 6

^{1}H NMR for 6-Nitro-3-(phenylsulfonyl)quinolin-2(1*H*)-one (**18**) 7
^{13}C NMR for 6-Nitro-3-(phenylsulfonyl)quinolin-2(1*H*)-one (**18**) 7

^{1}H NMR for 3-Carbethoxy-6-nitroquinolin-2(1*H*)-one (**19**) 8
^{13}C NMR for 3-Carbethoxy-6-nitroquinolin-2(1*H*)-one (**19**) 8

^{1}H NMR for 3-(4-Methoxyphenyl)-6-nitroquinolin-2(1*H*)-one (**20**) 9
^{13}C NMR for 3-(4-Methoxyphenyl)-6-nitroquinolin-2(1*H*)-one (**20**) 9

^{1}H NMR for 3-(2-Fluorophenyl)-6-nitroquinolin-2(1*H*)-one (**21**) 10
^{13}C NMR for 3-(2-Fluorophenyl)-6-nitroquinolin-2(1*H*)-one (**21**) 10

^{1}H NMR for 3-(4-Chlorophenyl)-6-nitroquinolin-2(1*H*)-one (**22**) 11
^{13}C NMR for 3-(4-Chlorophenyl)-6-nitroquinolin-2(1*H*)-one (**22**) 11

^{1}H NMR for 3-(2,5-Dimethylphenyl)-6-nitroquinolin-2(1*H*)-one (**23**) 12
^{13}C NMR for 3-(2,5-Dimethylphenyl)-6-nitroquinolin-2(1*H*)-one (**23**) 12

^{1}H NMR for 6-Cyano-3-phenylquinolin-2(1*H*)-one (**24**) 13
^{13}C NMR for 6-Cyano-3-phenylquinolin-2(1*H*)-one (**24**) 13

^{1}H NMR for 6-Cyano-3-(phenylsulfonyl)quinolin-2(1*H*)-one (**25**) 14
^{13}C NMR for 6-Cyano-3-(phenylsulfonyl)quinolin-2(1*H*)-one (**25**).......... 14

^{1}H NMR for 6-Carbomethoxy-3-phenylquinolin-2(1*H*)-one (**26**) 15
^{13}C NMR for 6-Carbomethoxy-3-phenylquinolin-2(1*H*)-one (**26**) 15

^{1}H NMR for 6-Carbomethoxy-3-(phenylsulfonyl)quinolin-2(1*H*)-one (**27**) 16
^{13}C NMR for 6-Carbomethoxy-3-(phenylsulfonyl)quinolin-2(1*H*)-one (**27**).................... 16

^{1}H NMR for 3-Phenyl-6-(trifluoromethyl)quinolin-2(1*H*)-one (**28**).................... 17
^{13}C NMR for 3-Phenyl-6-(trifluoromethyl)quinolin-2(1*H*)-one (**28**).................... 17

^{1}H NMR for 3-(Phenylsulfonyl)-6-(trifluoromethyl)quinolin-2(1*H*)-one (**29**).................... 18
^{13}C NMR for 3-(Phenylsulfonyl)-6-(trifluoromethyl)quinolin-2(1*H*)-one (**29**).................... 18

^{1}H NMR for 3-Phenyl-1,8-naphthyridin-2(1*H*)-one (**30**) 19
^{13}C NMR for 3-Phenyl-1,8-naphthyridin-2(1*H*)-one (**30**) 19

^{1}H NMR for 3-(Phenylsulfonyl)-1,8-naphthyridin-2(1*H*)-one (**31**) 20
^{13}C NMR for 3-(Phenylsulfonyl)-1,8-naphthyridin-2(1*H*)-one (**31**) 20

^{1}H NMR for 2-(2-Fluorophenyl)acetamide (14)

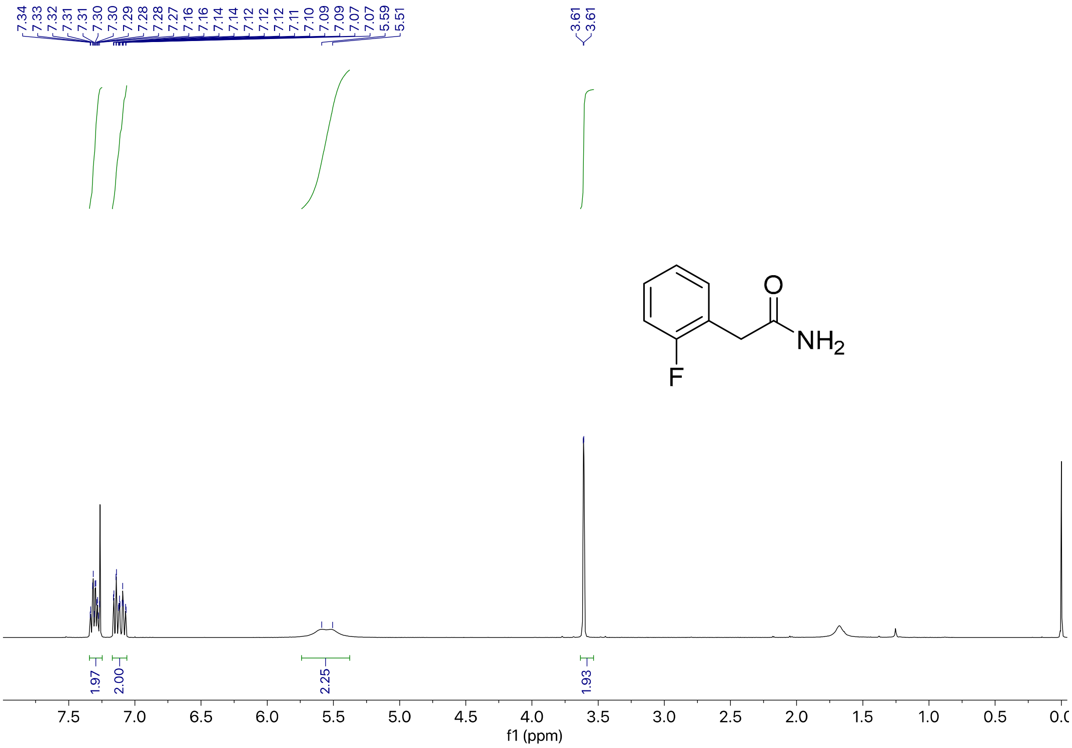

^{13}C NMR for 2-(2-Fluorophenyl)acetamide (14)

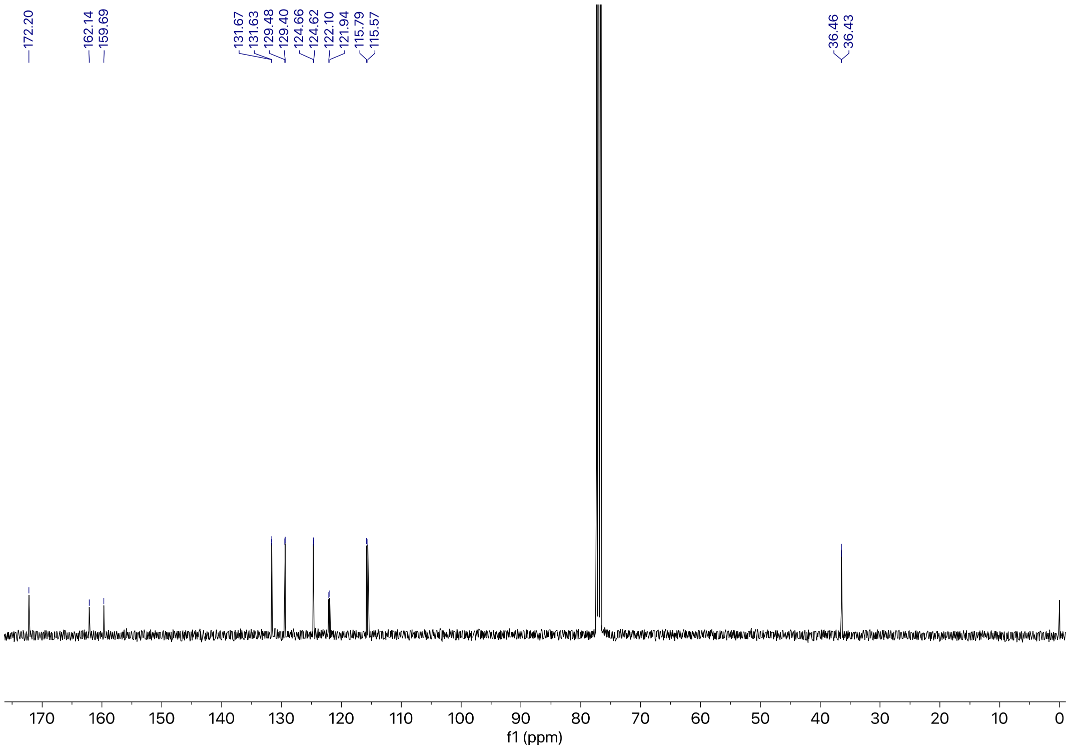

^{1}H NMR for 2-(4-Chlorophenyl)acetamide (15)

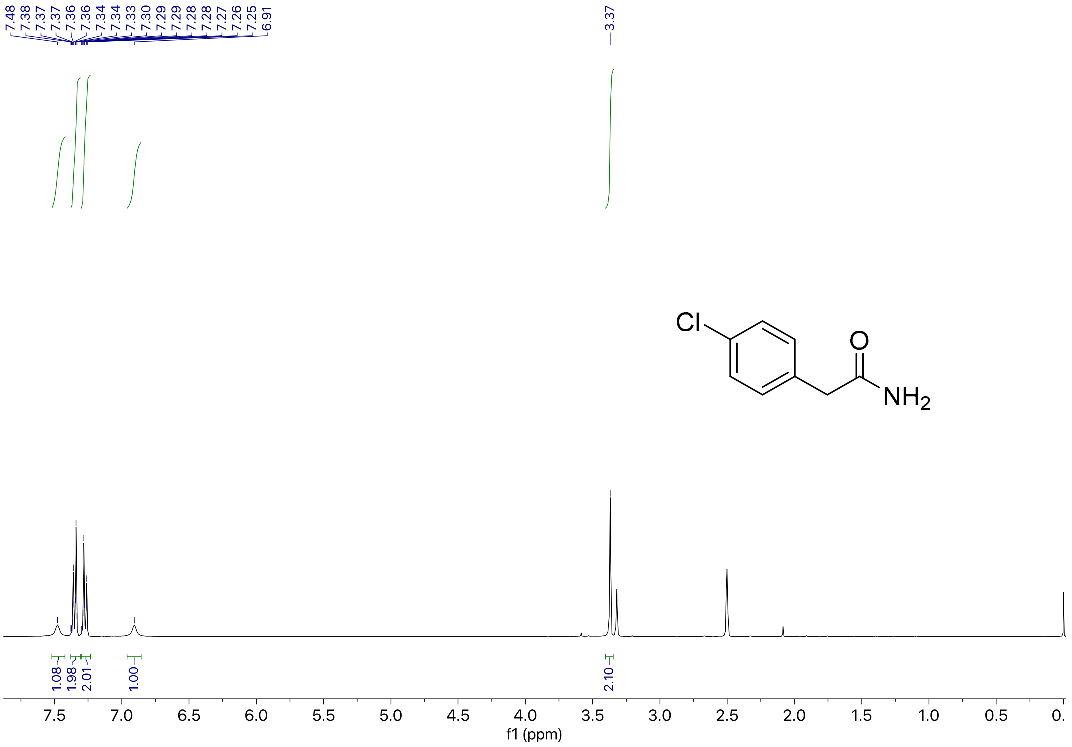

^{13}C NMR for 2-(4-Chlorophenyl)acetamide (15)

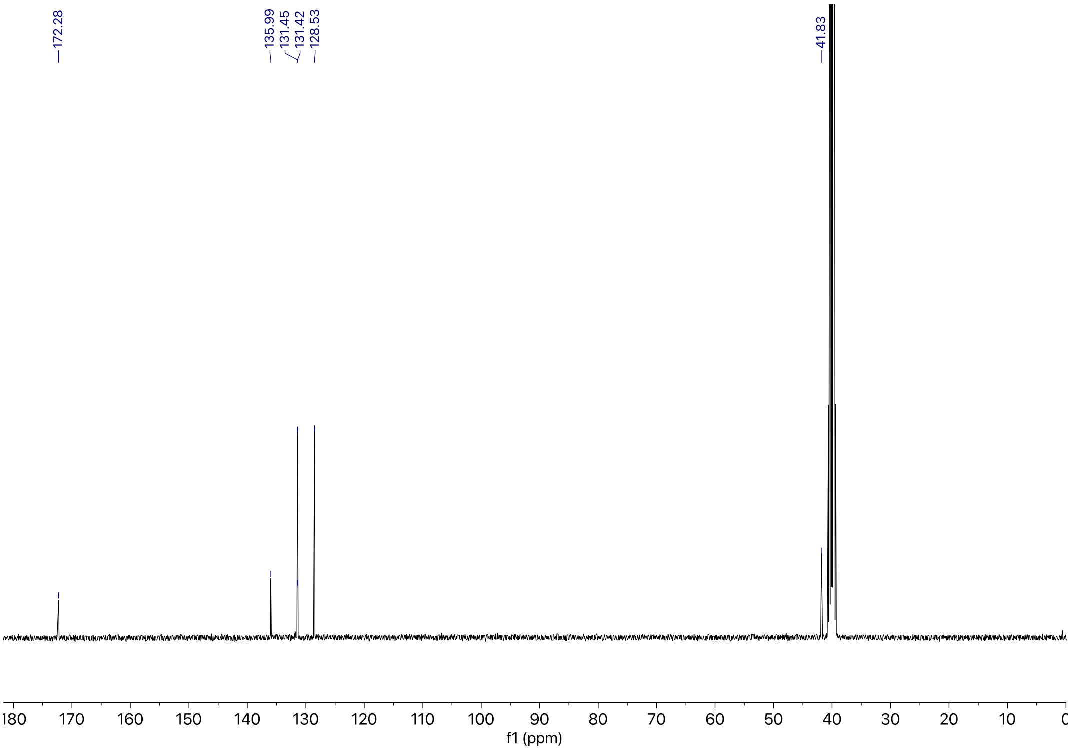

1H NMR for 2-(2,5-Dimethylphenyl)acetamide (16)

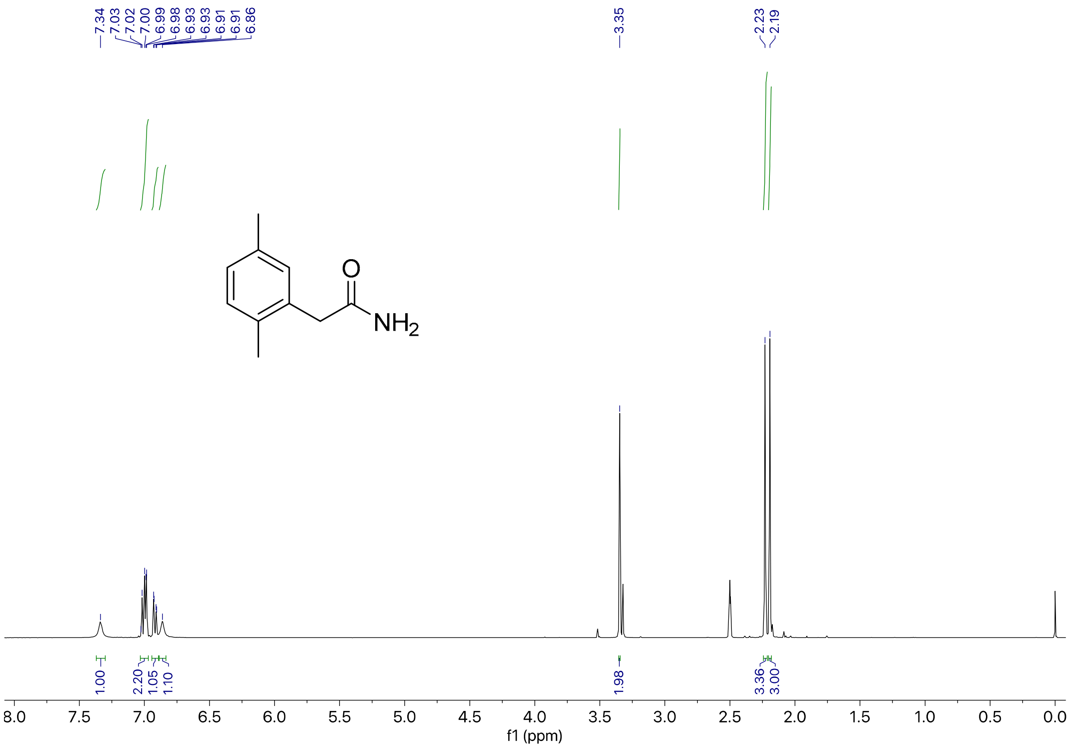

13C NMR for 2-(2,5-Dimethylphenyl)acetamide (16)

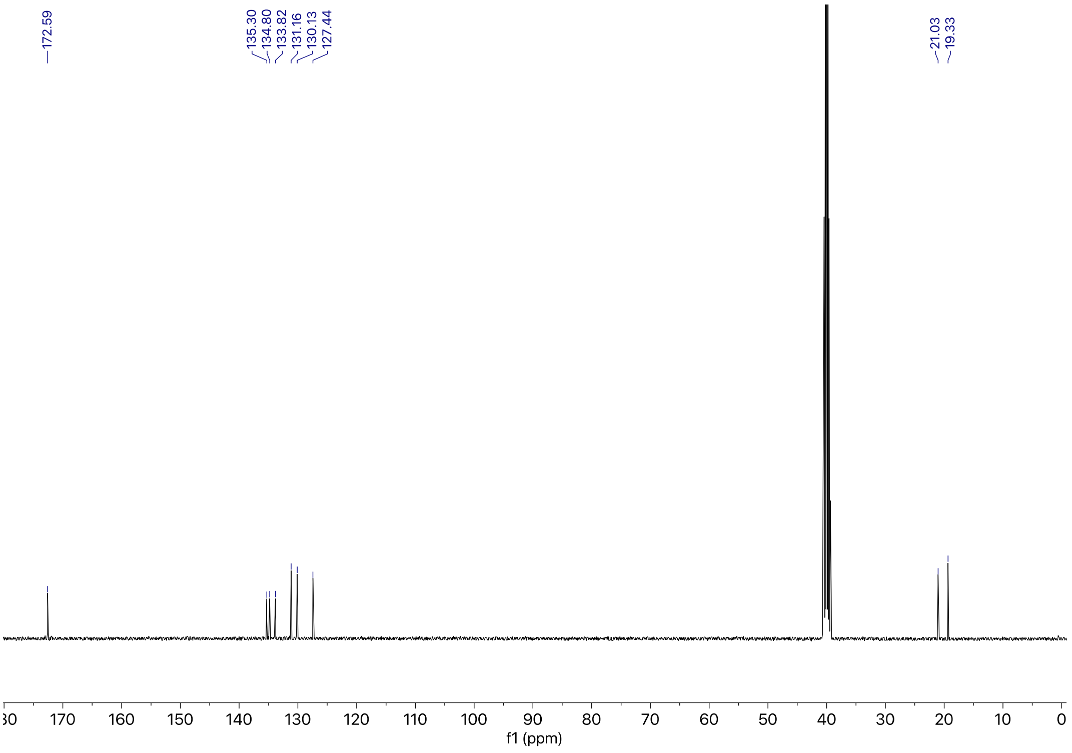

^{1}H NMR for 6-Nitro-3-phenylquinolin-2(1*H*)-one (17)

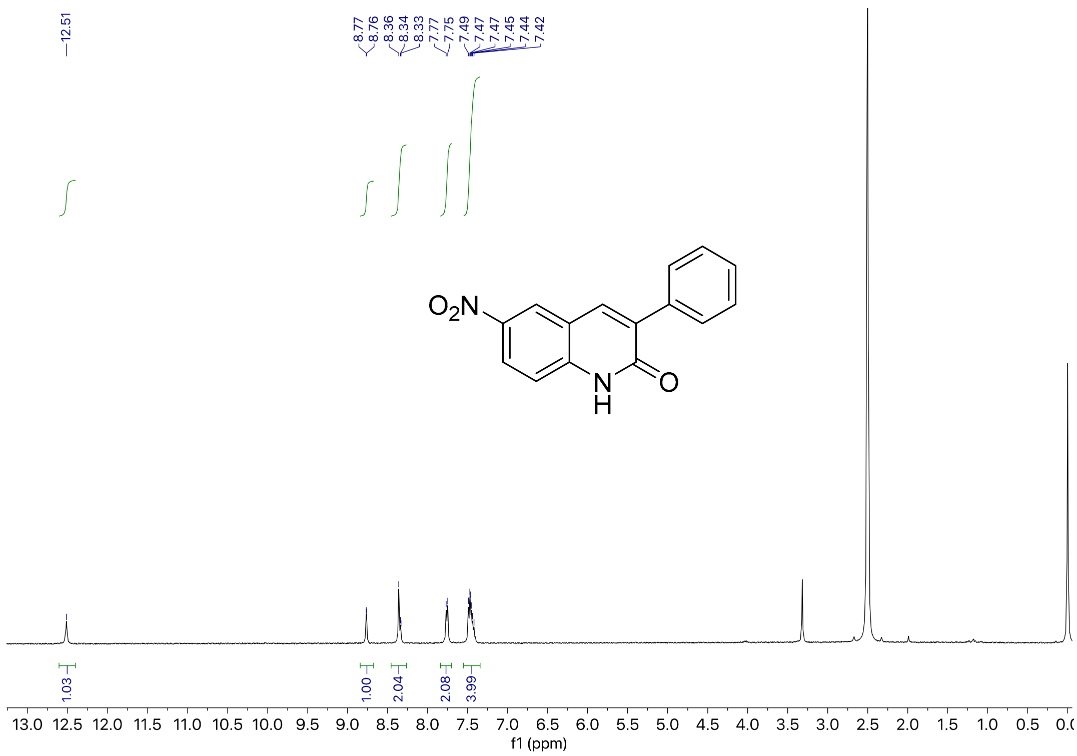

^{13}C NMR for 6-Nitro-3-phenylquinolin-2(1*H*)-one (17)

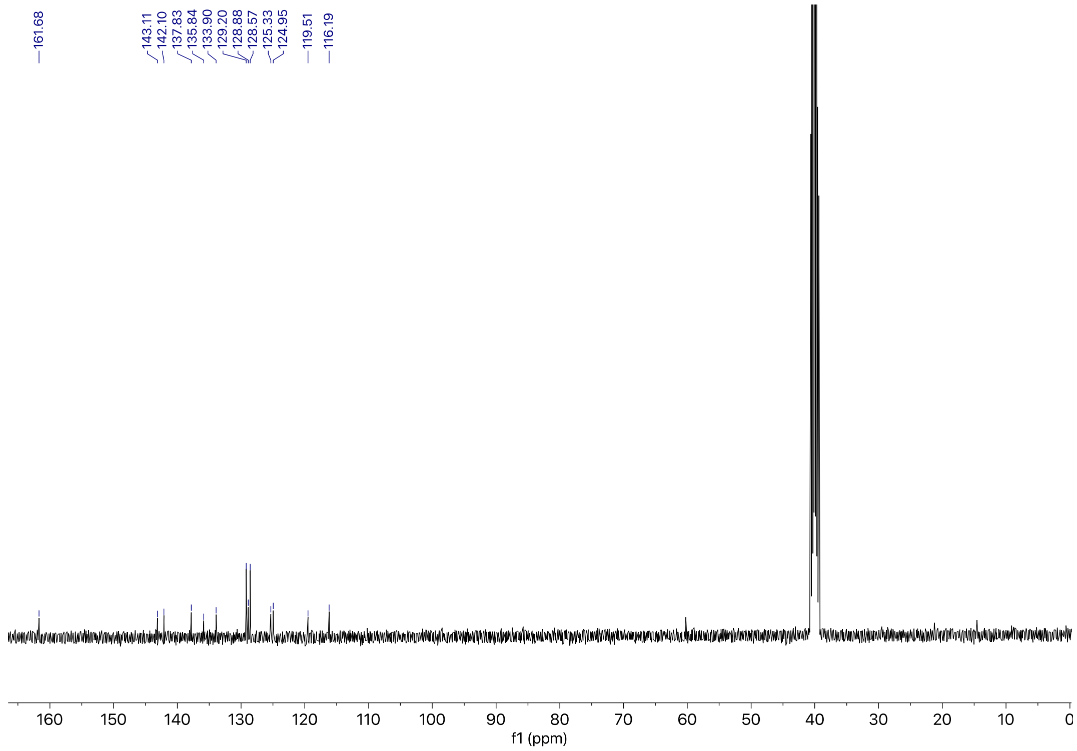

^{1}H NMR for 6-Nitro-3-(phenylsulfonyl)quinolin-2(1*H*)-one (18)

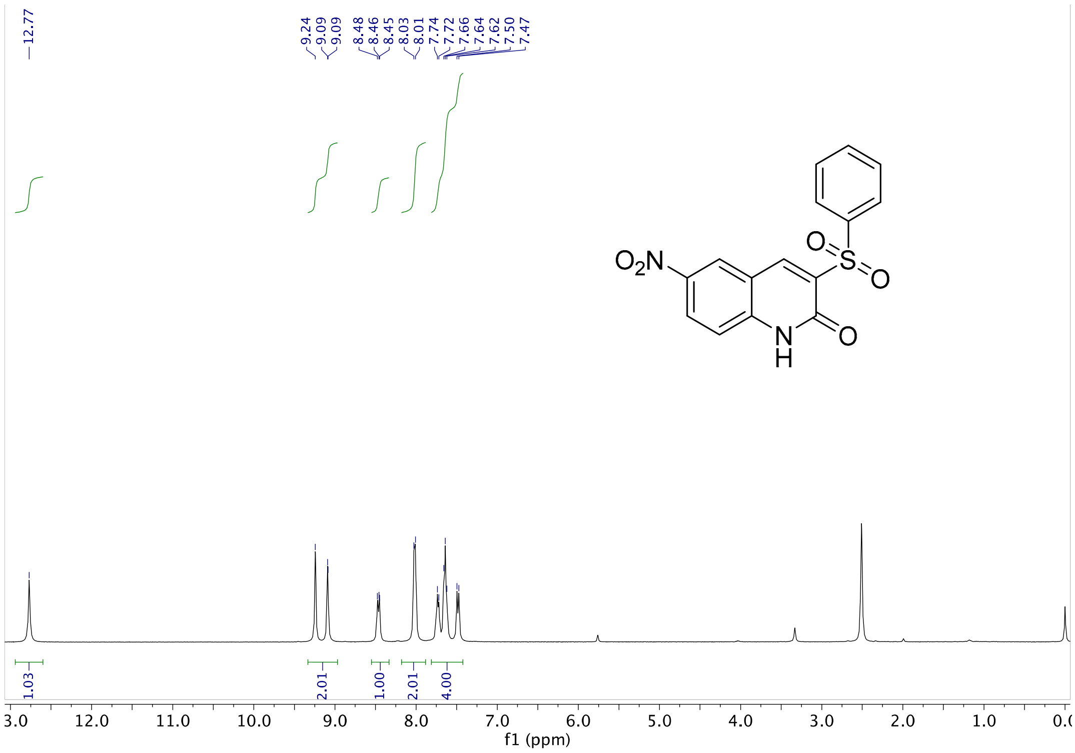

^{13}C NMR for 6-Nitro-3-(phenylsulfonyl)quinolin-2(1*H*)-one (18)

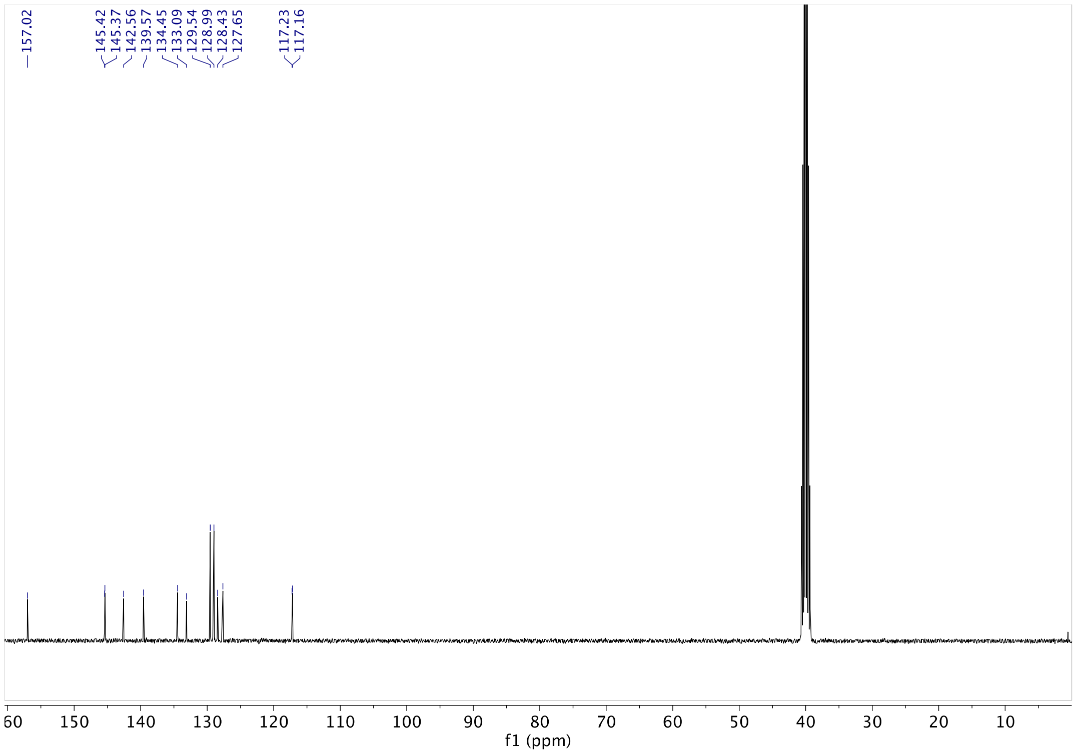

¹H NMR for 3-Carbethoxy-6-nitroquinolin-2(1*H*)-one (19)

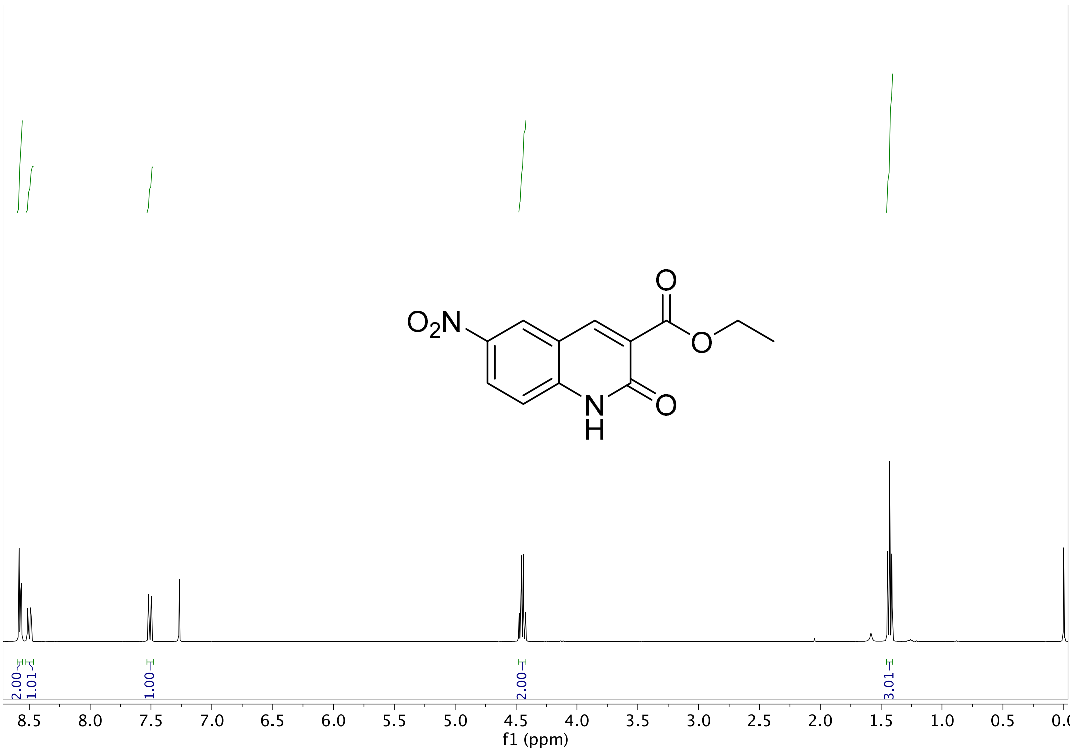

¹³C NMR for 3-Carbethoxy-6-nitroquinolin-2(1*H*)-one (19)

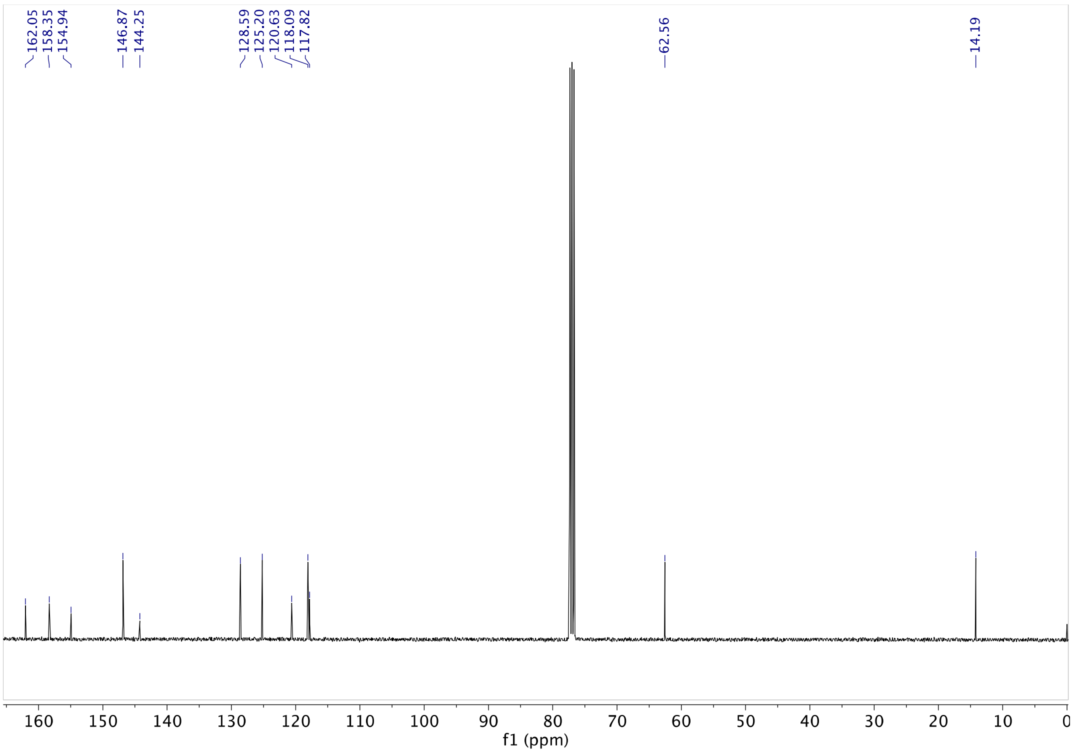

^{1}H NMR for 3-(4-Methoxyphenyl)-6-nitroquinolin-2(1*H*)-one (20)

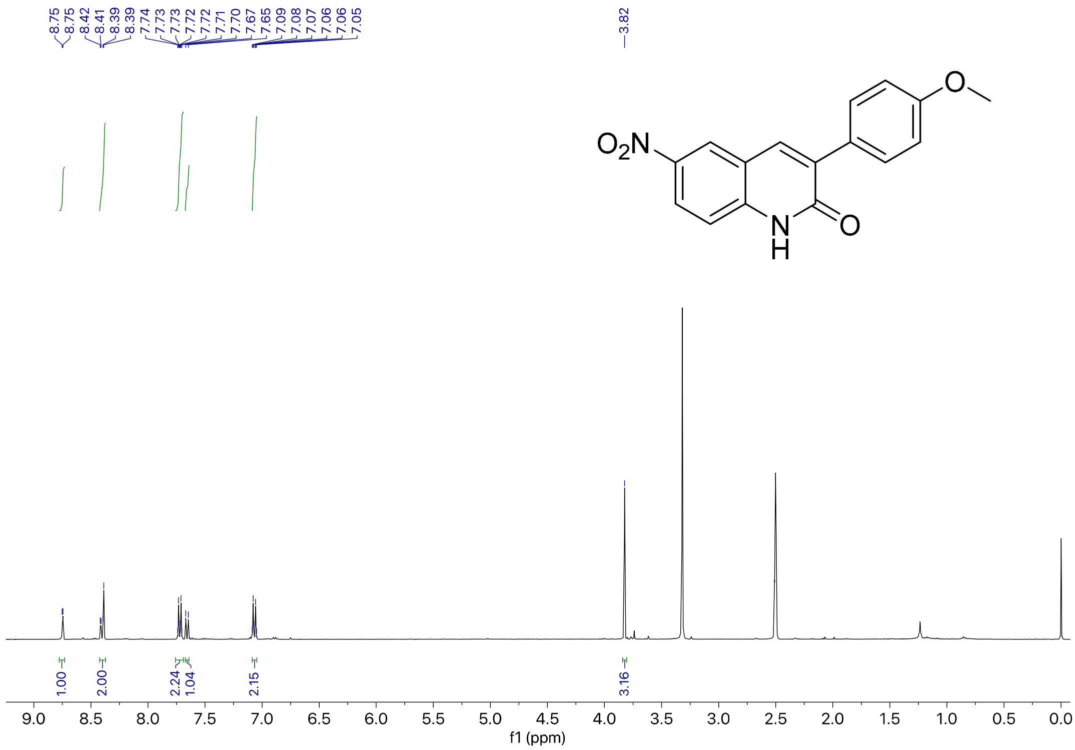

^{13}C NMR for 3-(4-Methoxyphenyl)-6-nitroquinolin-2(1*H*)-one (20)

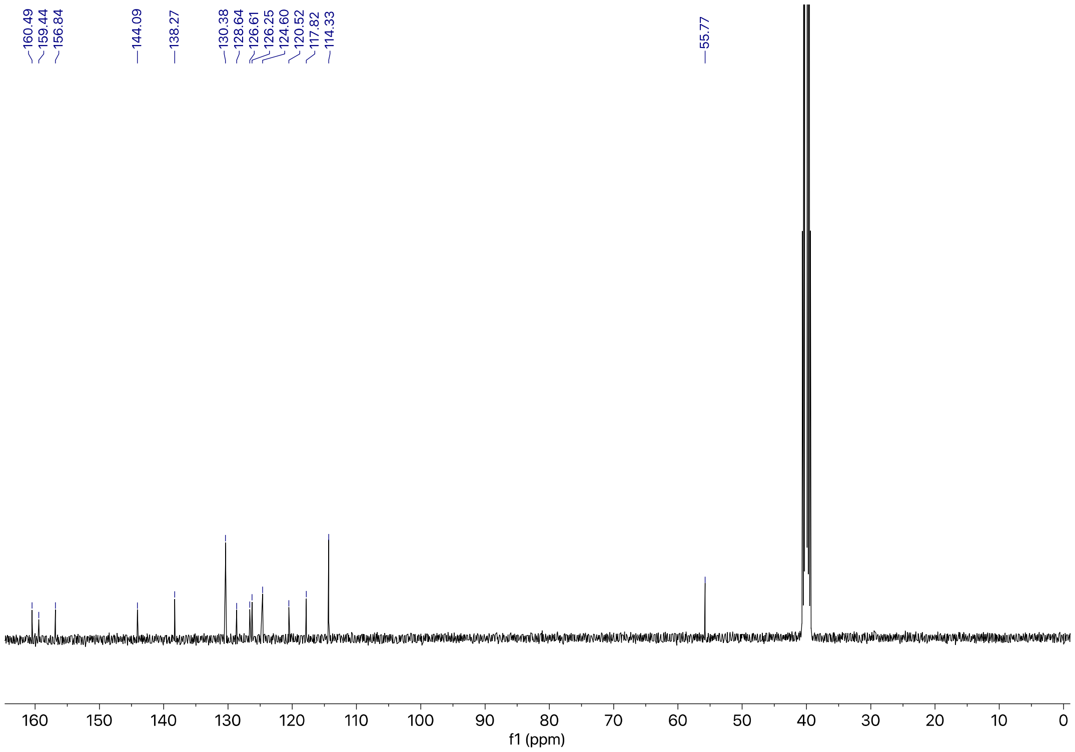

^{1}H NMR for 3-(2-Fluorophenyl)-6-nitroquinolin-2(1*H*)-one (21)

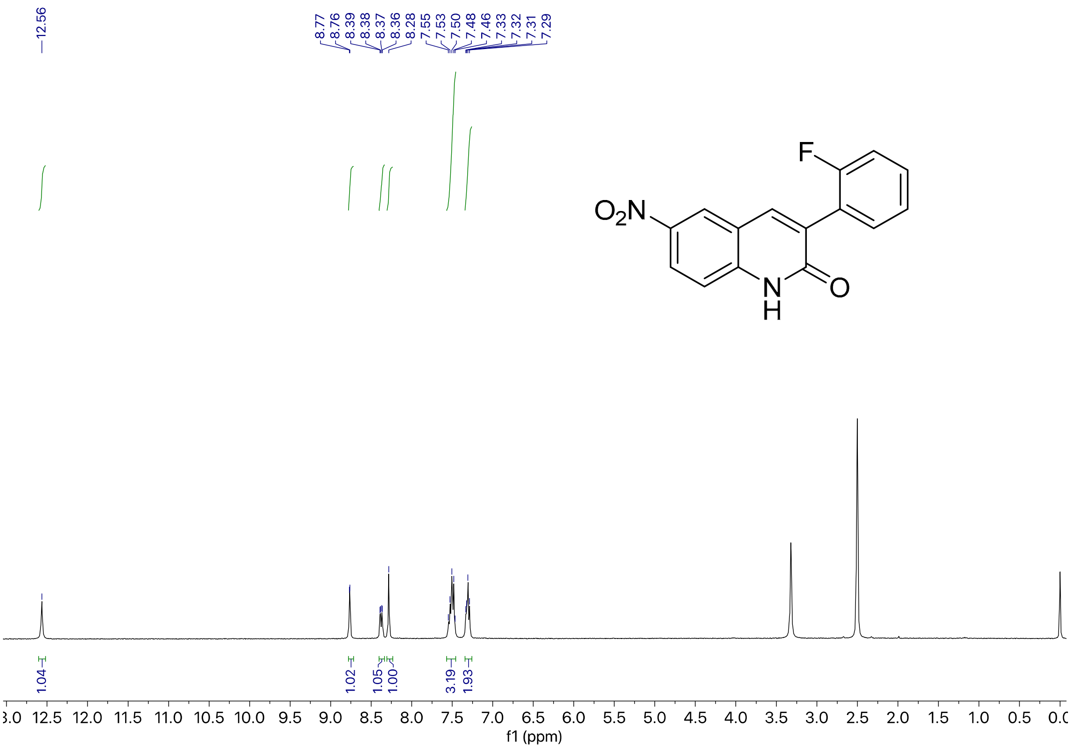

^{13}C NMR for 3-(2-Fluorophenyl)-6-nitroquinolin-2(1*H*)-one (21)

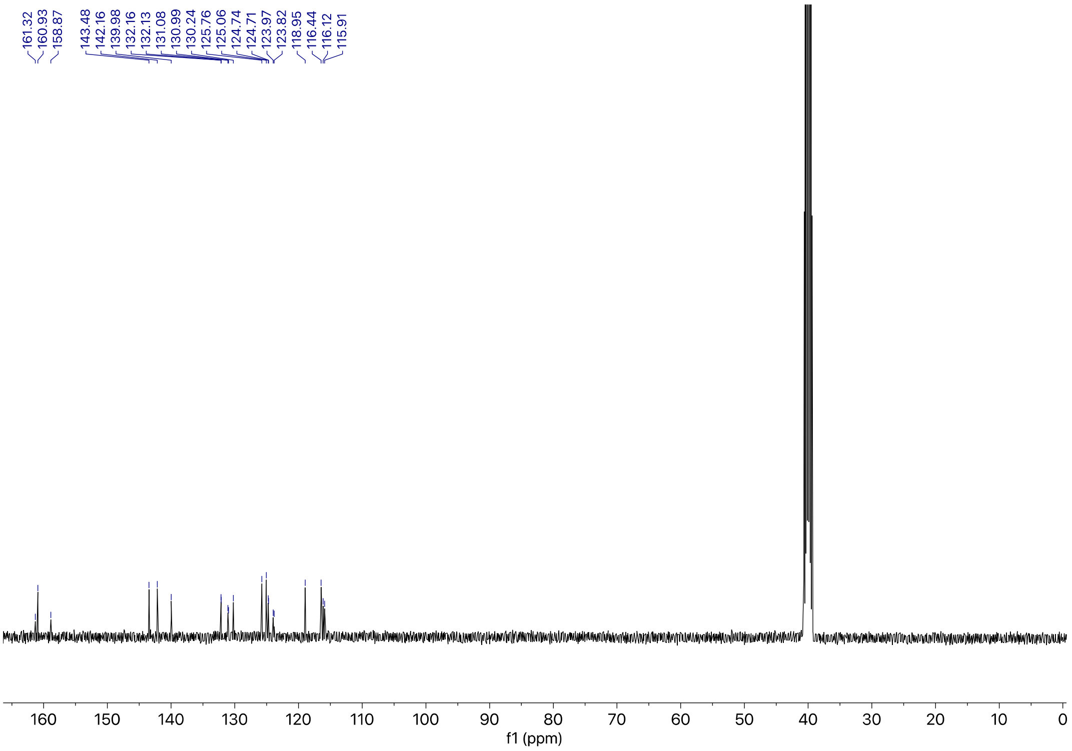

^{1}H NMR for 3-(4-Chlorophenyl)-6-nitroquinolin-2(1*H*)-one (22)

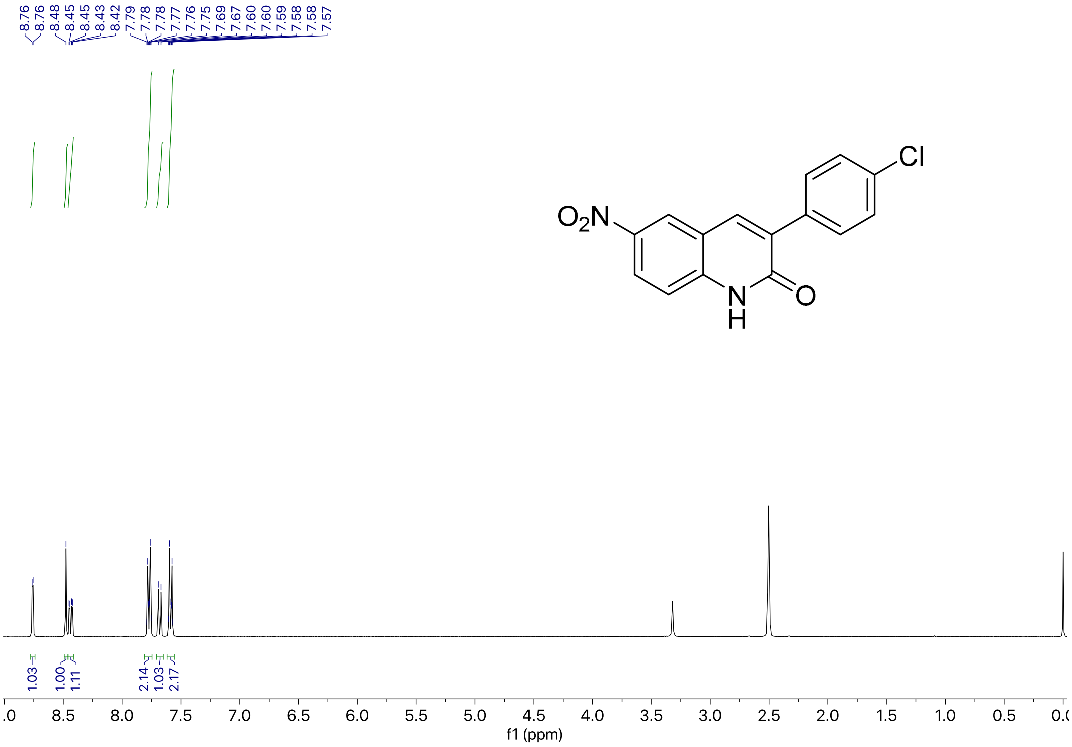

^{13}C NMR for 3-(4-Chlorophenyl)-6-nitroquinolin-2(1*H*)-one (22)

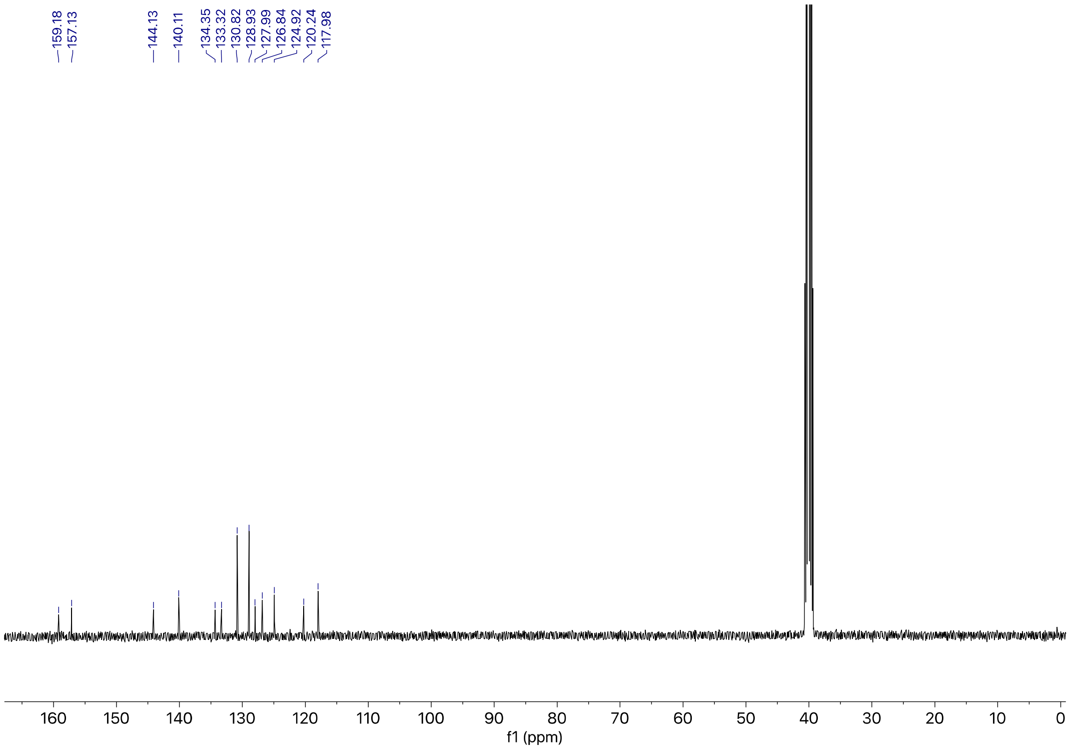

^{1}H NMR for 3-(2,5-Dimethylphenyl)-6-nitroquinolin-2(1*H*)-one (23)

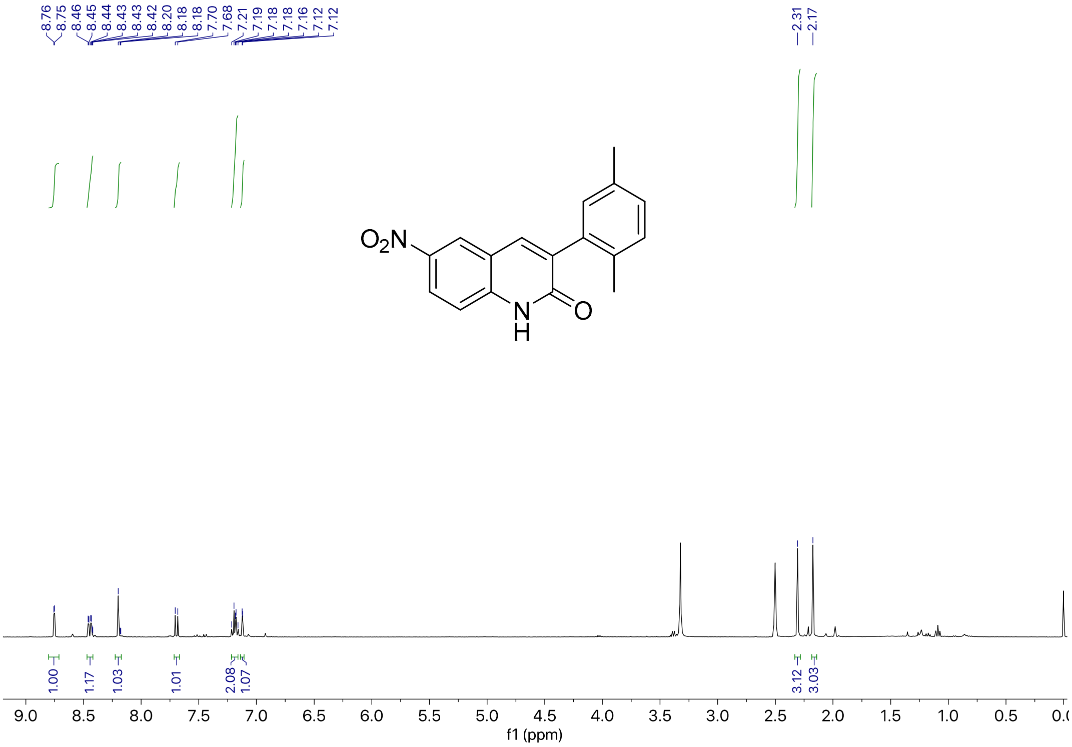

^{13}C NMR for 3-(2,5-Dimethylphenyl)-6-nitroquinolin-2(1*H*)-one (23)

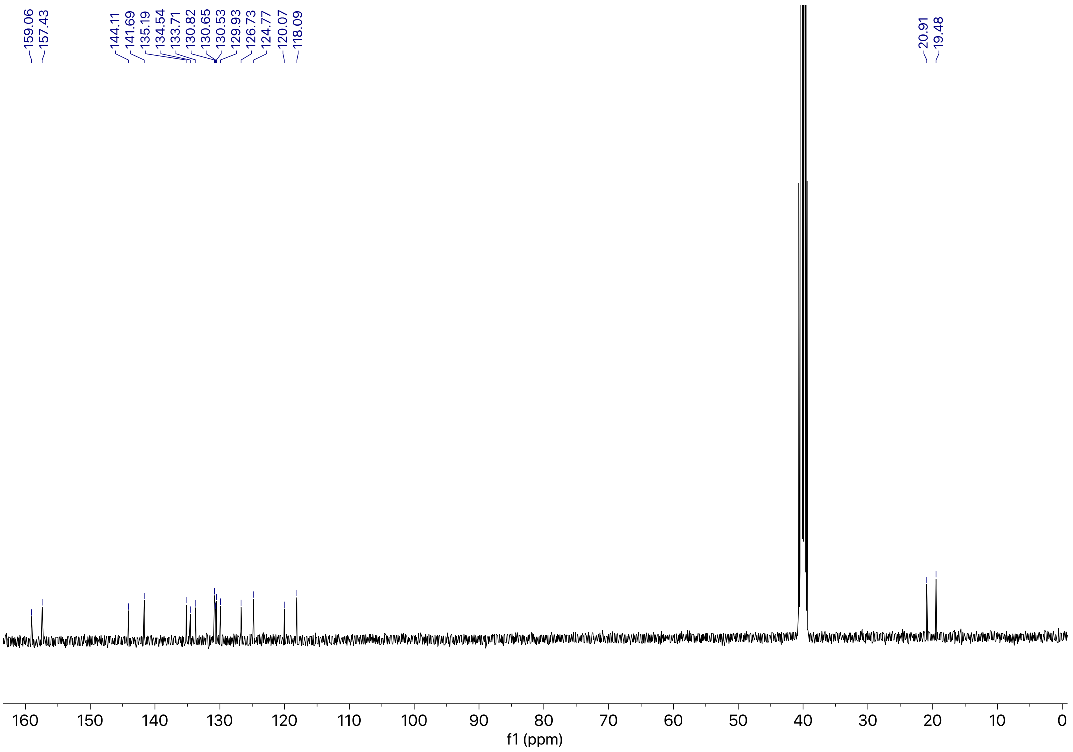

^{1}H NMR for 2-6-Cyano-3-phenylquinolin-2(1*H*)-one (24)

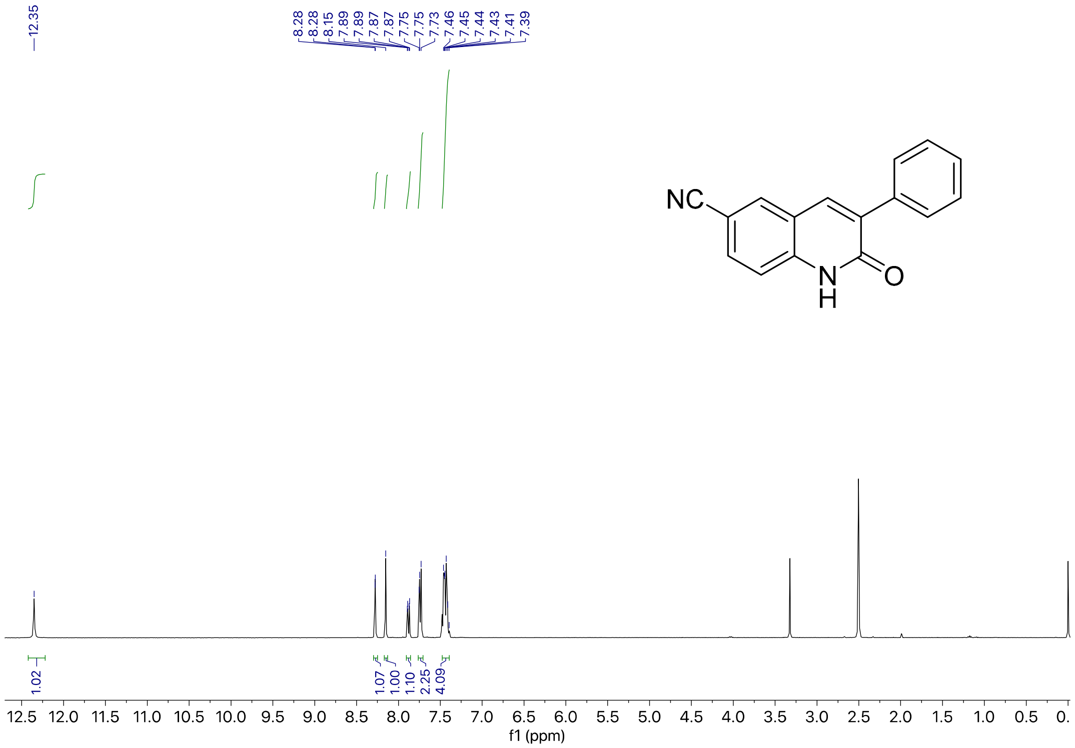

^{13}C NMR for 6-Cyano-3-phenylquinolin-2(1*H*)-one (24)

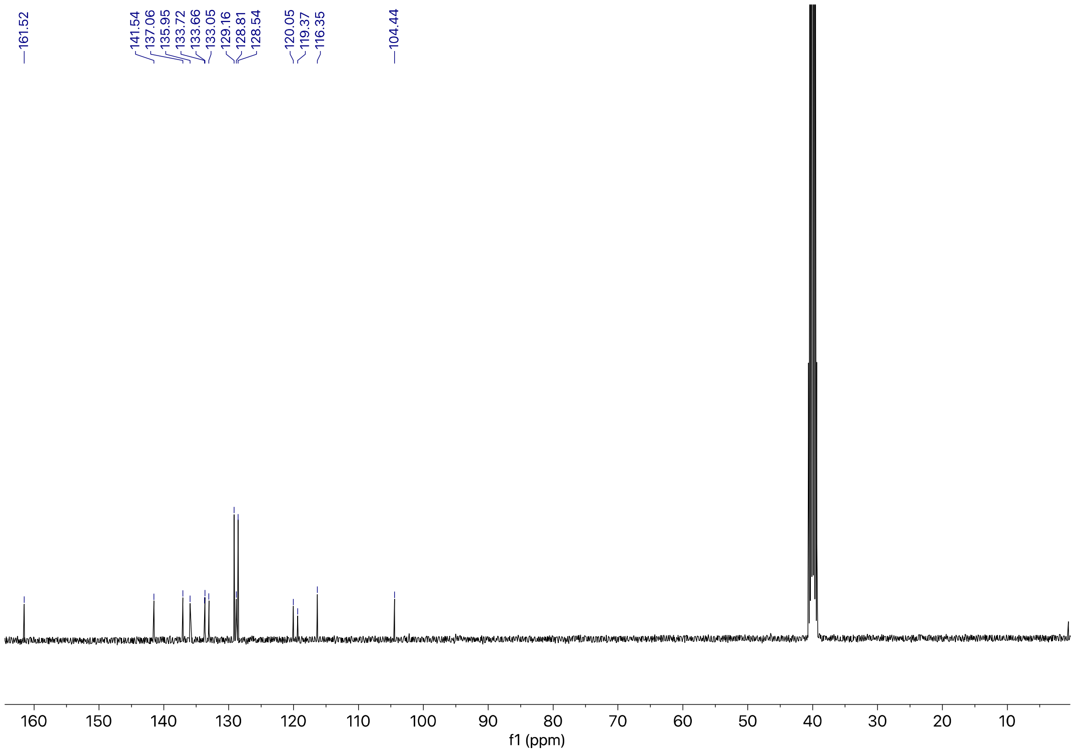

^1H NMR for 6-Cyano-3-(phenylsulfonyl)quinolin-2(1*H*)-one (25)

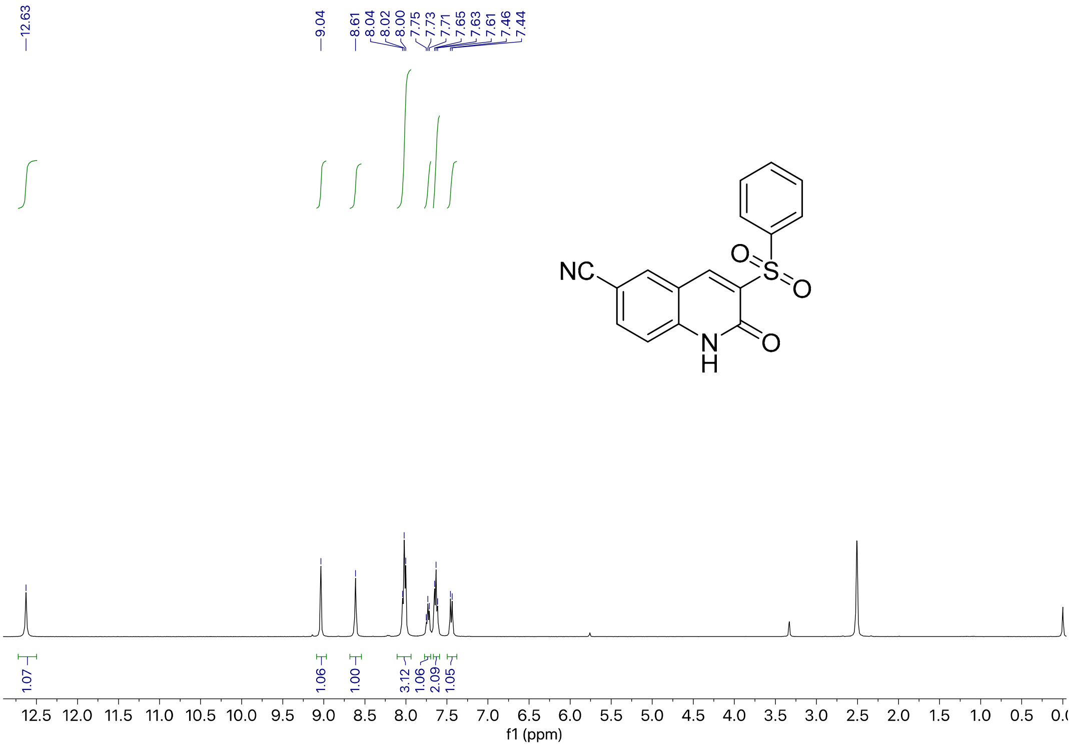

^{13}C NMR for 6-Cyano-3-(phenylsulfonyl)quinolin-2(1*H*)-one (25)

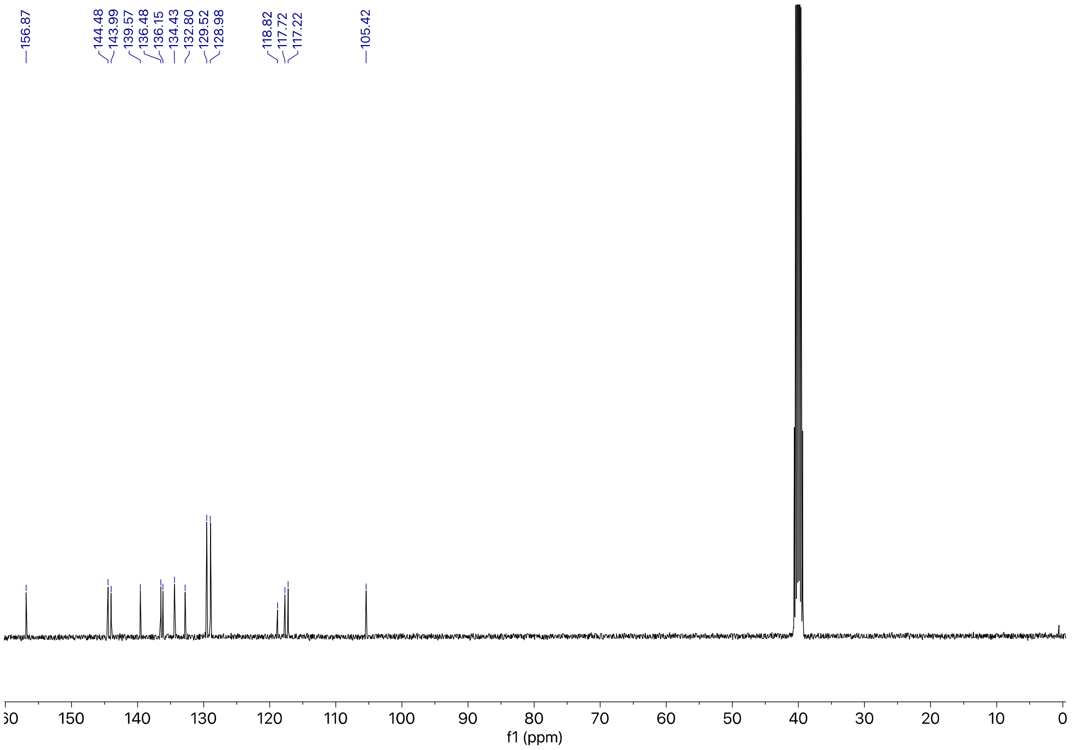

¹H NMR for 6-Carbomethoxy-3-phenylquinolin-2(1*H*)-one (26)

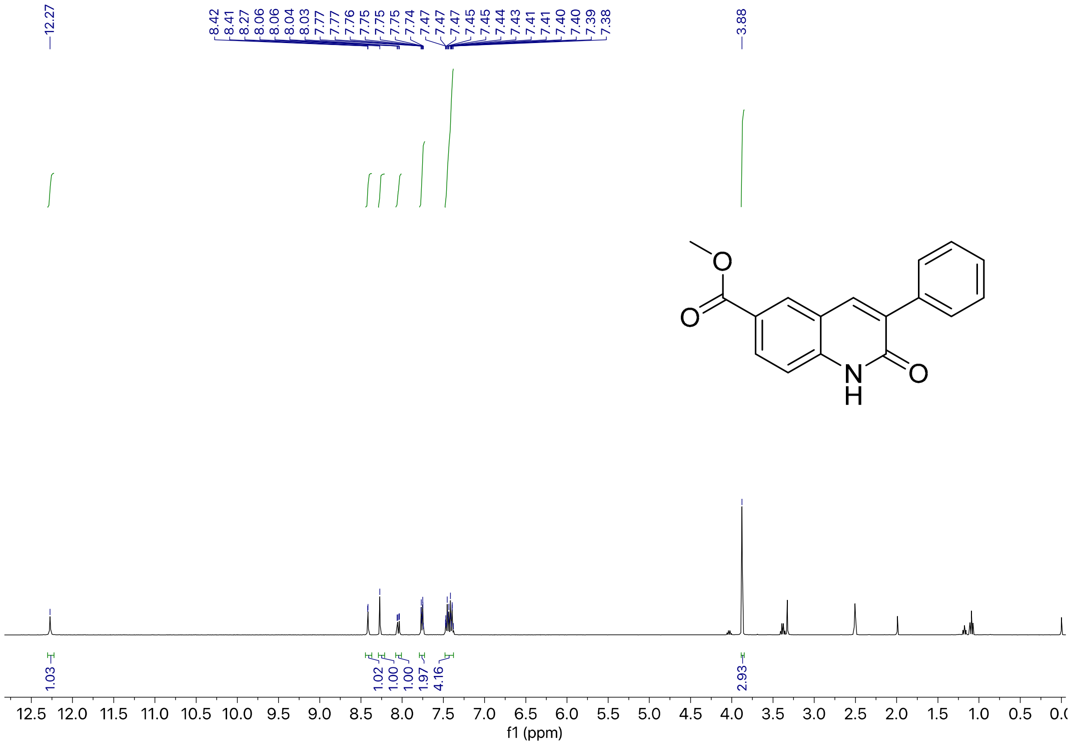

¹³C NMR for 6-Carbomethoxy-3-phenylquinolin-2(1*H*)-one (26)

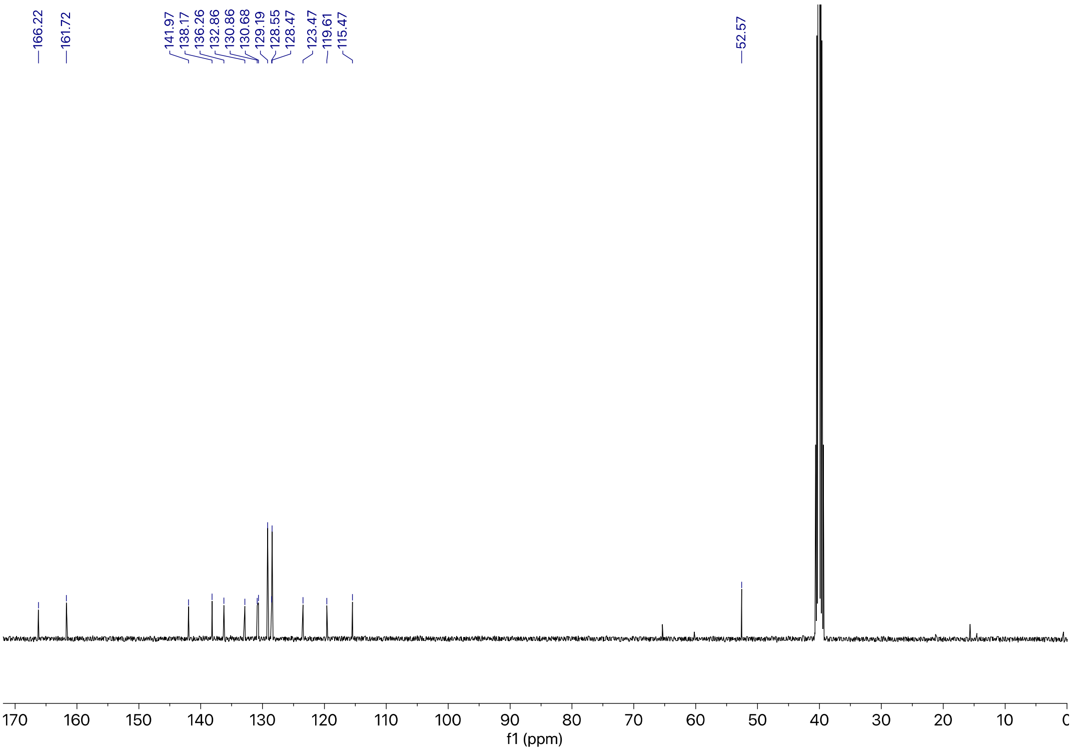

^{1}H NMR for 6-Carbomethoxy-3-(phenylsulfonyl)quinolin-2(1*H*)-one (27)

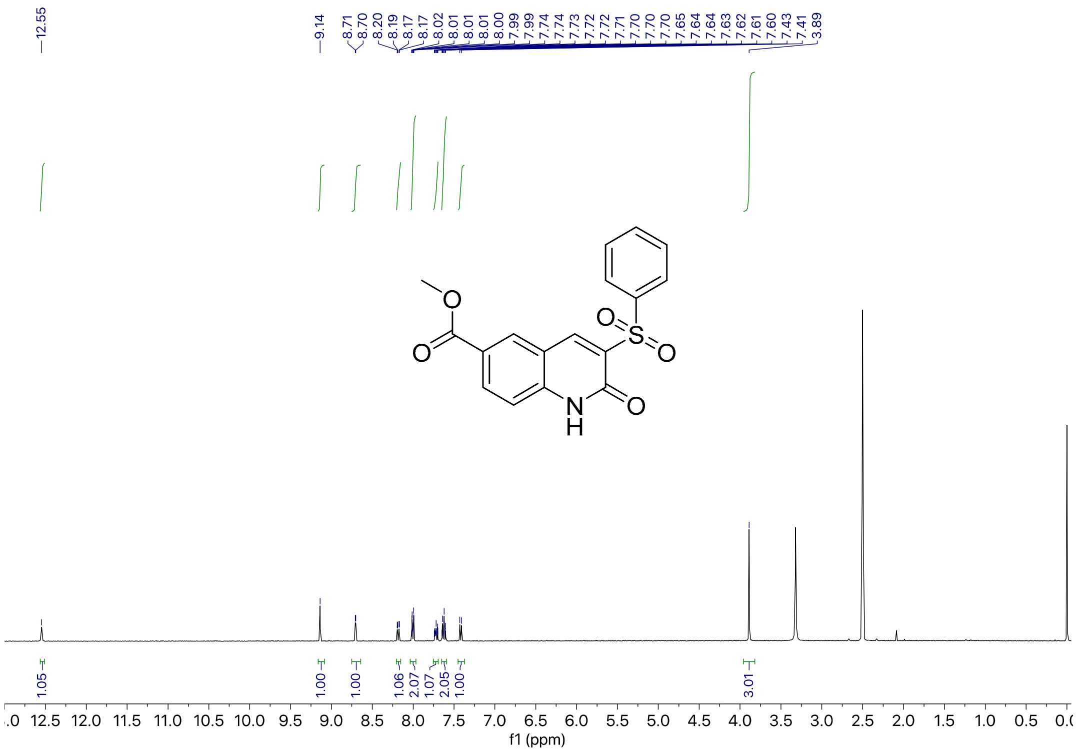

^{13}C NMR for 6-Carbomethoxy-3-(phenylsulfonyl)quinolin-2(1*H*)-one (27)

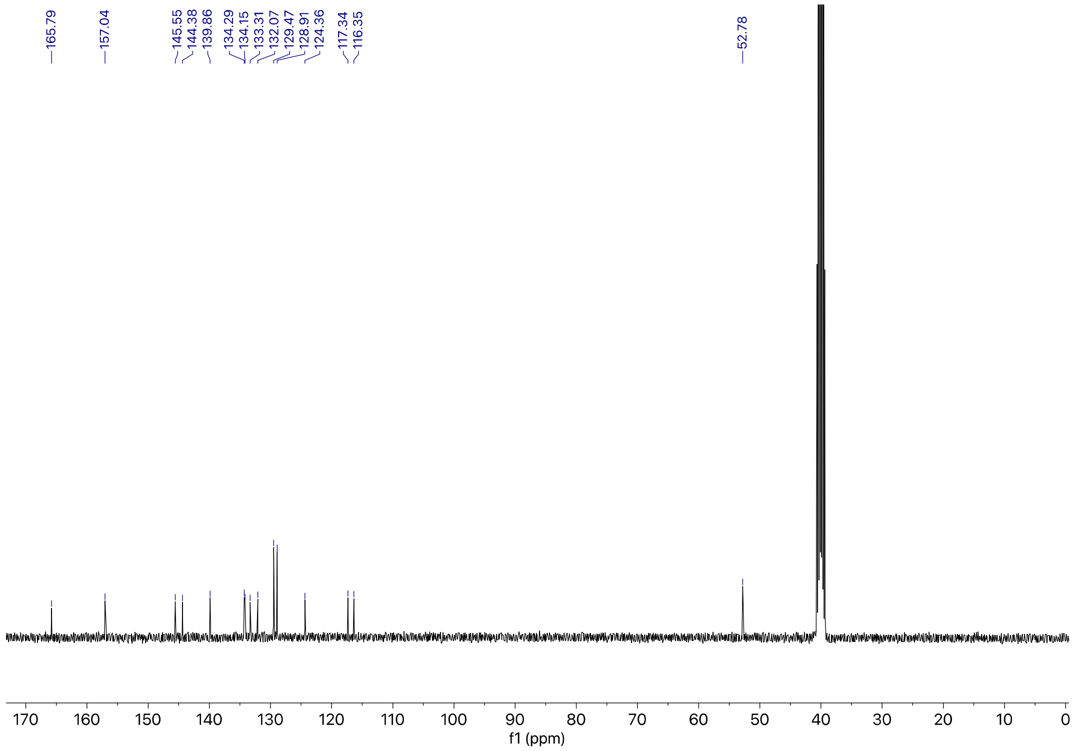

^{1}H NMR for 3-Phenyl-6-(trifluoromethyl)quinolin-2(1*H*)-one (28)

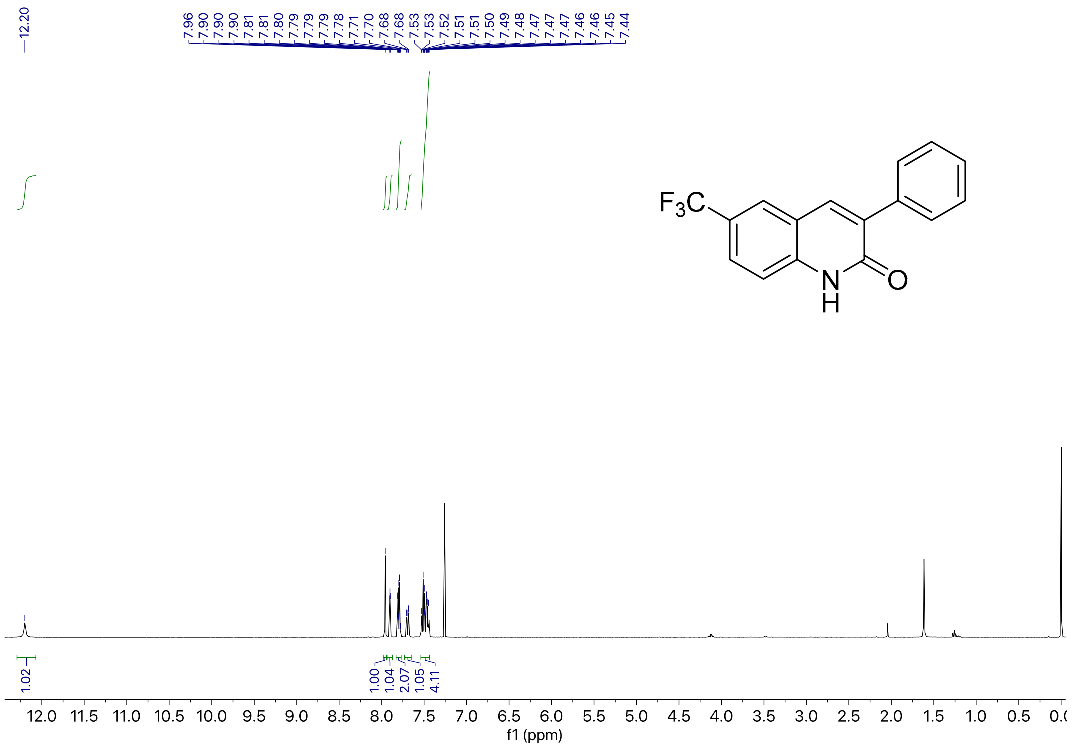

^{13}C NMR for 3-Phenyl-6-(trifluoromethyl)quinolin-2(1*H*)-one (28)

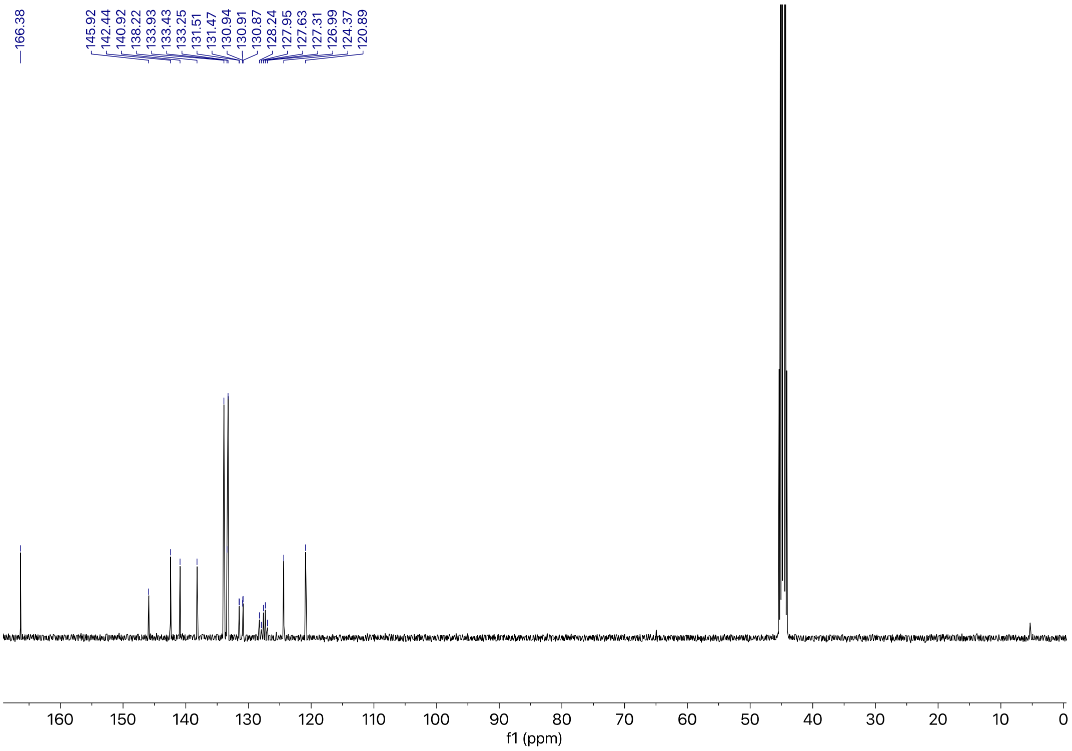

^{1}H NMR for 3-(Phenylsulfonyl)-6-(trifluoromethyl)quinolin-2(1*H*)-one (29)

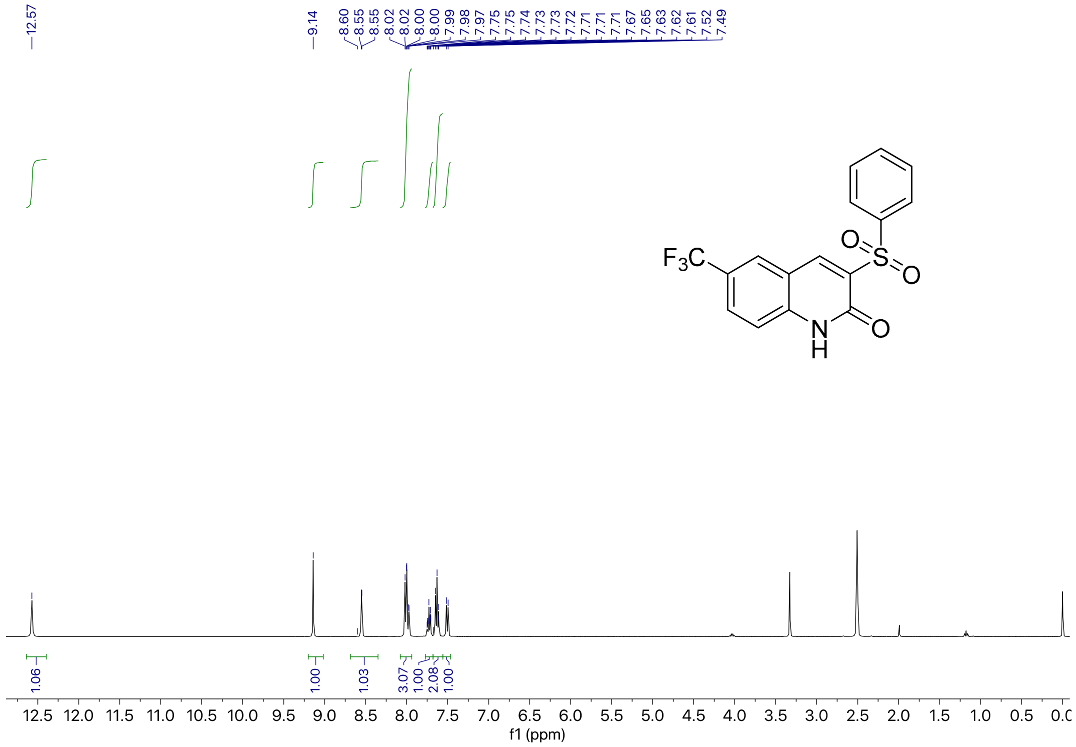

^{13}C NMR for 3-(Phenylsulfonyl)-6-(trifluoromethyl)quinolin-2(1*H*)-one (29)

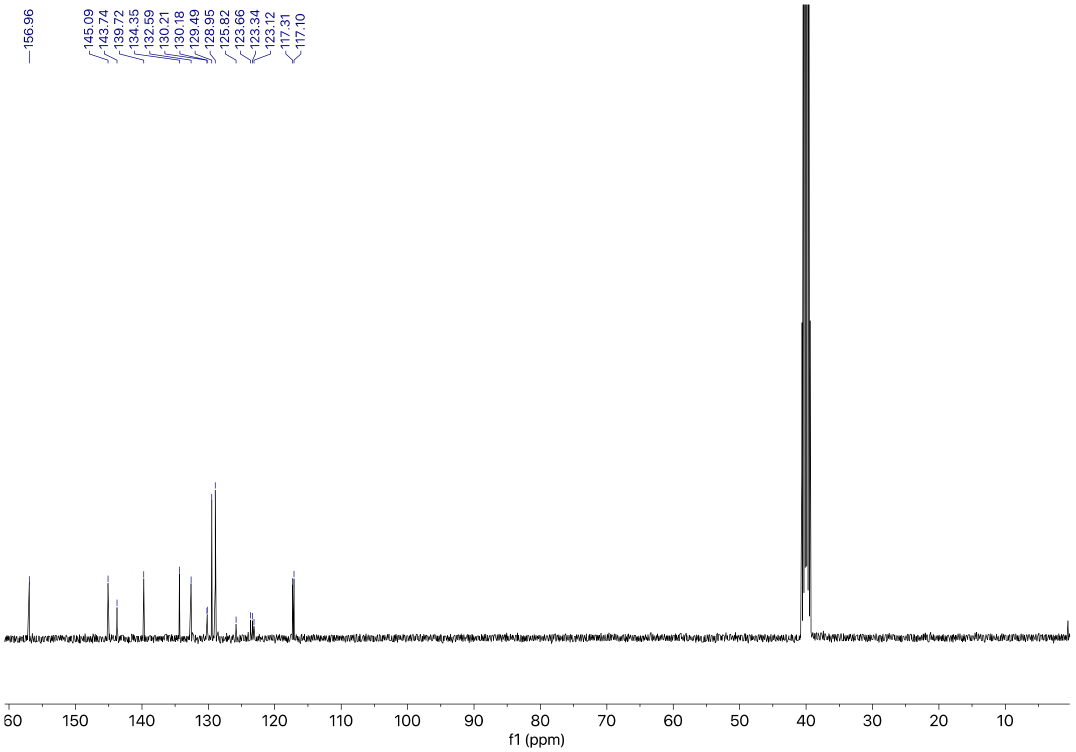

^{1}H NMR for 3-Phenyl-1,8-naphthyridin-2(1*H*)-one (30)

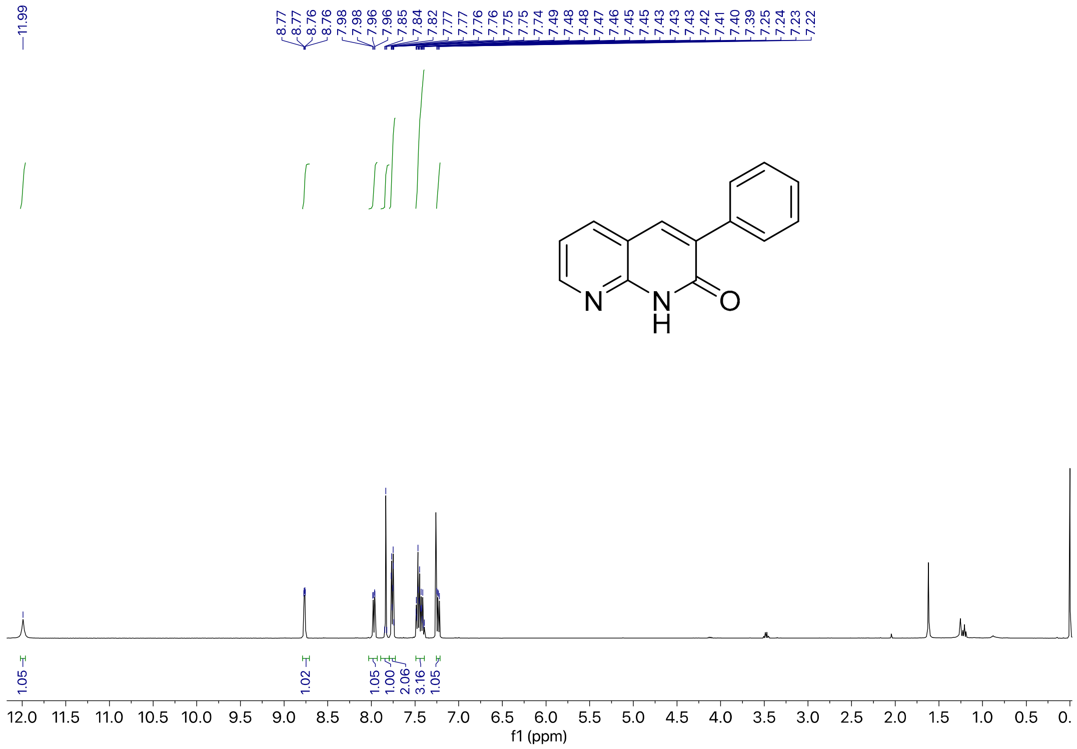

^{13}C NMR for 3-Phenyl-1,8-naphthyridin-2(1*H*)-one (30)

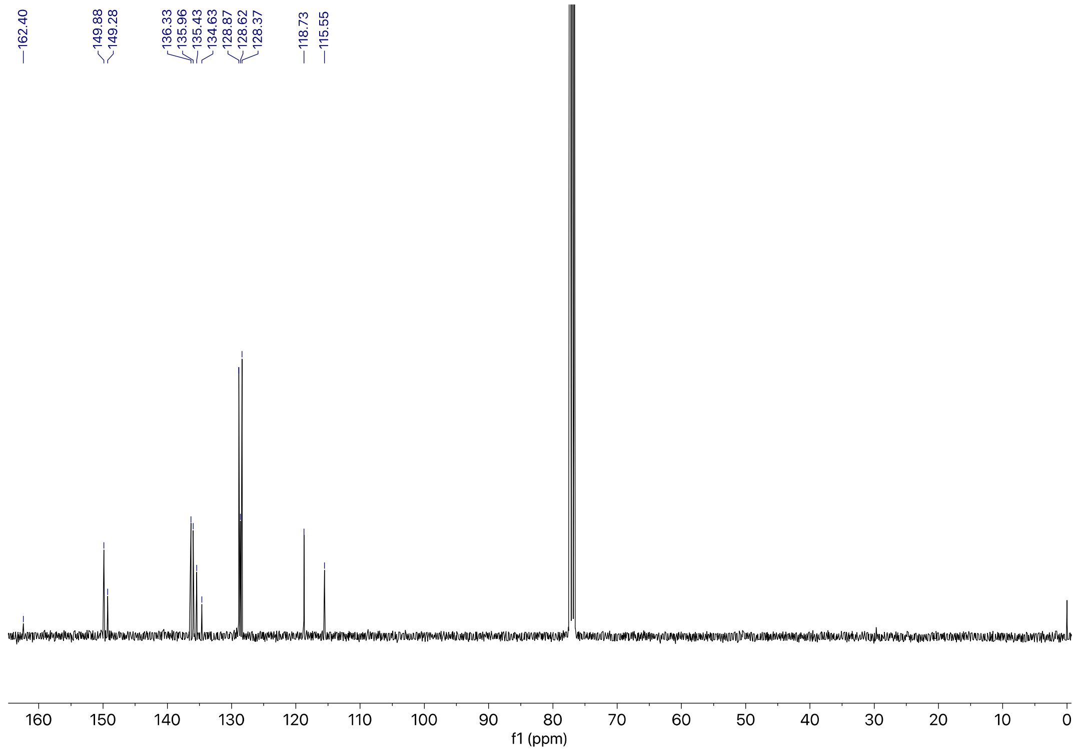

^{1}H NMR for 3-(Phenylsulfonyl)-1,8-naphthyridin-2(1*H*)-one (31)

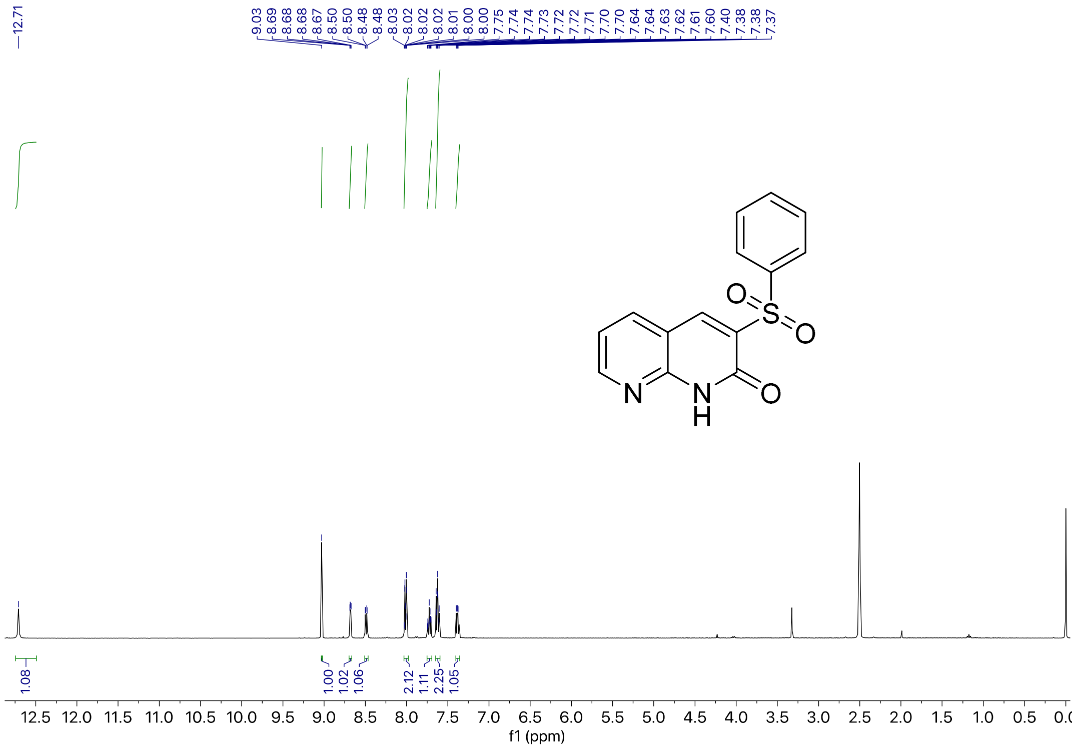

^{13}C NMR for 3-(Phenylsulfonyl)-1,8-naphthyridin-2(1*H*)-one (31)

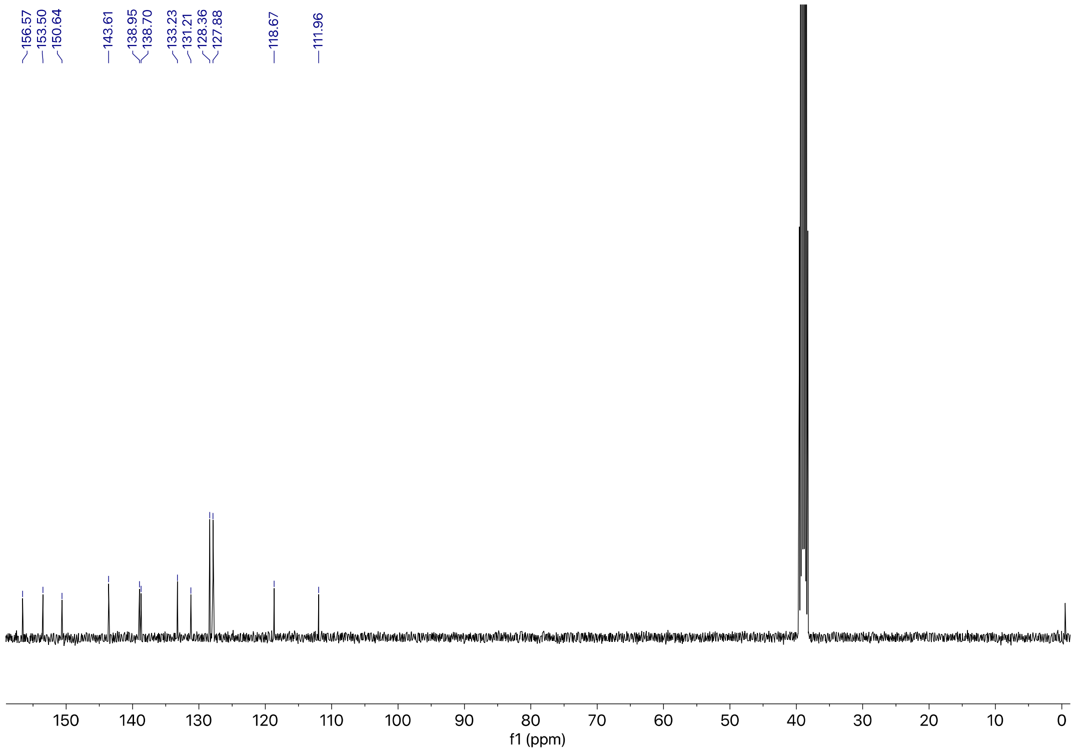

Supplement: Supplementary file 1 [file molecules-28-05856-s001.zip › molecules-2511341-supplementary.pdf]
